# Phosphoproteomic Landscaping Identifies Non-canonical cKIT Signaling in Polycythemia Vera Erythroid Progenitors

**DOI:** 10.3389/fonc.2019.01245

**Published:** 2019-11-22

**Authors:** Giulia Federici, Lilian Varricchio, Fabrizio Martelli, Mario Falchi, Orietta Picconi, Federica Francescangeli, Paola Contavalli, Gabriella Girelli, Agostino Tafuri, Emanuel F. Petricoin, Maria Mazzarini, Ann Zeuner, Anna Rita Migliaccio

**Affiliations:** ^1^IRCCS Regina Elena National Cancer Institute, Rome, Italy; ^2^Tisch Cancer Institute, Icahn School of Medicine at Mount Sinai, New York, NY, United States; ^3^National Center for Preclinical and Clinical Research and Evaluation of Pharmaceutical Drugs, Istituto Superiore di Sanità, Rome, Italy; ^4^National HIV/AIDS Center, Istituto Superiore di Sanità, Rome, Italy; ^5^Oncology and Molecular Medicine, Istituto Superiore di Sanità, Rome, Italy; ^6^Immunohematology and Transfusion Medicine Unit, “La Sapienza” University of Rome, Rome, Italy; ^7^Sant'Andrea Hospital—La Sapienza, Department of Clinic and Molecular Medicine “La Sapienza” University of Rome, Rome, Italy; ^8^Center for Applied Proteomics and Molecular Medicine, George Mason University, Manassas, VA, United States; ^9^Department of Biomedical and Neuromotorial Sciences, Alma Mater University, Bologna, Italy; ^10^Myeloproliferative Neoplasm Research Consortium, Division of Hematology and Medical Oncology, Icahn School of Medicine at Mount Sinai, New York, NY, United States

**Keywords:** stem cell factor, cKIT, erythropoiesis, polycythemia vera, erythroid progenitors

## Abstract

Although stem cell factor (SCF)/cKIT interaction plays key functions in erythropoiesis, cKIT signaling in human erythroid cells is still poorly defined. To provide new insights into cKIT-mediated erythroid expansion in development and disease, we performed phosphoproteomic profiling of primary erythroid progenitors from adult blood (AB), cord blood (CB), and Polycythemia Vera (PV) at steady-state and upon SCF stimulation. While AB and CB, respectively, activated transient or sustained canonical cKIT-signaling, PV showed a non-canonical signaling including increased mTOR and ERK1 and decreased DEPTOR. Accordingly, screening of FDA-approved compounds showed increased PV sensitivity to JAK, cKIT, and MEK inhibitors. Moreover, differently from AB and CB, in PV the mature 145kDa-cKIT constitutively associated with the tetraspanin CD63 and was not endocytosed upon SCF stimulation, contributing to unrestrained cKIT signaling. These results identify a clinically exploitable variegation of cKIT signaling/metabolism that may contribute to the great erythroid output occurring during development and in PV.

## Introduction

Great levels of erythroid expansion are associated with fetal development (1) and Polycythemia Vera (PV), a Philadelphia-negative myeloproliferative neoplasm characterized by erythrocytosis (2–4). The mechanisms underlying such expansions are subjected to extensive investigation because of their clinical relevance. On one hand, the discovery of ontogenetic-specific regulatory mechanisms may lead to the identification of novel erythroid stimulating agents. On the other, mechanisms active in PV may unveil new therapeutic approaches for this disease. In fact, although PV patients usually respond well to phlebotomy, alternative more aggressive treatments are often warranted to control splenomegaly and/or thrombosis and therapies to prevent disease progression to acute leukemia have not been devised as yet (3).

The majority of PV patients harbor the gain-of-function *JAK2*V617F mutation (5–7) that potentiates EPO-R signaling (8). Therefore, by contrast with normal cells, erythroid progenitor cells from PV grow *in vitro* without EPO (9, 10). This discovery raised hope that JAK inhibitors may be effective treatments for PV. Unfortunately, the clinical trials published up-to-now revealed that these drugs ameliorate symptoms but do not alter the natural history of PV (11) prompting continuous search for additional therapeutic strategies. In addition to EPO-R, JAK2 is constitutively associated to cKIT (12) and erythroid progenitors from PV are more sensitive than normal cells to cKIT inhibitors Imatinib (13) and Dasatinib (14). Clinical trials with similar tyrosine kinase inhibitors reported some efficacy in PV (15, 16), but only in some of the patients enrolled. A deeper understanding of cKIT signaling in human erythroid cells would facilitate the identification of novel therapeutic targets for this disease (16).

cKIT is the receptor for stem cell factor (SCF) and controls the proliferation and maturation of healthy erythroid progenitors. These effects are exerted in combination with EPO-R in an ontogeny-specific fashion (17). In fact, murine fetal erythroid progenitors remain dependent on SCF up to terminal maturation while adult cells shift from a prevalent SCF-dependent to an EPO-dependent state as they mature (18, 19). The biochemistry of this synergy resides in the physical association between cKIT tyrosine(Y)567 (murine Y568) (20) and the intracytoplasmic box2 domain of EPO-R (21) which sustain proliferation of murine cell lines in response to SCF (22, 23). Unfortunately, information on cKIT signaling and metabolism during the ontogenesis of human erythroid cells is scanty. The aim of this study was to build a comprehensive landscape of cKIT signaling in human erythroid cells during ontogenesis and in *JAK2*V617F-PV. Inspired by pioneer studies highlighting the importance of global signaling studies in erythroid and leukemic cells (24–28), we investigated cKIT signaling by comparing the phosphoproteomic profiles of erythroid cells generated *ex-vivo* from PV, cord blood (CB, a source of fetal cells) and adult blood (AB).

## Experimental Procedures

### Human Subjects

Low volume CB (*n* = 26), buffy coats from regular blood donations (*n* = 37) and blood from JAK2V617F-PVpatients (*n* = 25, allele burden >67–90%) who underwent phlebotomy as part of their treatment were provided as de-identified material according to guidelines established by institutional ethical committees for human subject studies as recommended by the 1975 Helsinki Declaration revised in 2000. This study does not require IRB approval because it uses only human biological samples already collected and stored in tissue banks. Mononuclear and CD14negCD34pos cells (>98% pure by FACS re-analyses) were isolated as described (29).

### Human Erythroid Massive Amplification Culture (HEMA)

CD14negCD34pos cells (10^4^ cells/mL) were cultured for 10 days in HEMA with SCF (100 ng/mL, Amgen, Thousand Oaks, CA), EPO (3 U/mL, Janssen, Raritan, NJ), IL-3 (10 ng/mL, RD System, Minneapolis, MN), dexamethasone (Dex, 10^−6^ M) and estradiol (10^−6^ M) (Sigma) (30).

### Cell Number and Phenotypic Analysis

Cell numbers and viability were assessed by microscopic evaluation after trypan blue staining (Boston Bioproducts, Ashland, MA). Phenotypic analysis was performed by flow-cytometry using Fluorescein Thiocyanate (FITC)–conjugated CD36 (31), phycoerythrin (PE)–conjugated CD235a, Phycoerythrin-Cyanin5.5 (PE-Cy5.5)-CD117 (cKIT) and Allophycocyanin (APC)-conjugated CD63 (BioLegend, San Diego, CA) or appropriate isotype controls (all from Becton Dickinson Biosciences, Franklin Lakes, NJ). Fluorescence intensities were measured with FACS ARIA (Becton Dickinson Biosciences). Dead cells were excluded by Sytox Blue staining.

### Reverse-Phase Protein Array (RPPA) Analysis

RPPA were constructed and analyzed as described (28). Arrays were probed with a library of ~180 antibodies. Acquired images were analyzed with MicroVigene v5.0. (VigeneTech, Carlisle, MA) for spot detection, local background subtraction, negative control subtraction, replicate averaging and total protein normalization. The software package JMP v6 (SAS Institute, Cary, NC) was used for internal standardization, two-way hierarchical clustering using Wards method, and two-groups Wilcoxon test (significance cut off *p* ≤ 0.05). Data are available at http://capmm.gmu.edu/data.

### Western Blot (WB) and Immunoprecipitation (IP)

Cell extracts (30 μg protein/lane) were separated on SDS-PAGE and transferred to nitrocellulose membranes which were incubated with appropriate primary and horseradish peroxidase-coupled secondary (Calbiochem, San Diego, CA) antibodies (32). Immune complexes were detected with the enhanced chemiluminescence kit (Amersham, Buckinghamshire, UK). For IP, cell extracts (300–500 μg) were incubated with anti-cKIT or anti-CD63 antibodies overnight at 4°C and then with Ultralink Immobilized Protein A/G sepharose (Pierce Biotech, Rockford, IL) for 2 h. Immune complexes were dissociated by boiling the beads for 5′ in loading buffer, separated on SDS-PAGE and analyzed by WB with various antibodies (32).

### Confocal Microscopy

Cells were cytospined on polylysine-coated glass slides, fixed in 4% paraformaldehyde and permeabilized in Triton X-100 (0.1%) (Bio-Rad Laboratories, Richmond, CA), incubated overnight at 4°C with primary antibodies, washed twice in PBS and incubated with Alexa Fluor-conjugated secondary antibodies (30′, room temperature), stained for 15′ with Dapi (Invitrogen) and mounted with Prolong-Gold antifade (Invitrogen). Slides were analyzed on a FV1000 confocal microscope (Olympus, Tokyo, Japan) equipped with a 60X oil immersion objective. Images were analyzed with the open source software ImageJ (https://imagej.nih.gov/ij/).

### Proliferation Assays

Day-10 erythroid cells (10^5^ cells/100 μL/well) were cultured for 24 h with increasing concentration of SCF alone or with IL-3+EPO+Dex (GFs) and/or cKIT neutralizing antibodies (R&D system, Minneapolis, USA). After 24 h (3-(4,5-dimethylthiazolyl-2)-2,5-diphenyltetrazolium bromide (MTT, 5 mg/mL, Sigma), was added and colorimetric reactions read as optical density (OD) at 570 nm (Victor3TM 1420 Multilabel Counter, Perkin Elmer, Waltham, MA). Proliferation with FDA-approved inhibitors of cell signaling from the Selleck Chemicals library (Houston, TX, USA) and in response to shRNA-treatment was evaluated with the CellTiter-Glo Luminescent Cell Viability Assay (Promega, Madison, WI). In this assay, erythroid cells (5–10 × 10^3^ cells/100 μL) were cultured for 24 h and then incubated with the CellTiter-Glo reagent, as described by the manufacture. Luminescence was recorded using VictorTMX3 multilabel plate reader (excitation 560 nm, emission 590 nm).

### shRNA Experiments

Day-7 erythroid cells (2 × 10^6^cells) were transfected with pGIPZ vectors containing GFP alone, GFP-tagged scrambled-shRNA or GFP-tagged CD63-shRNA (RHS4531 GE Healthcare Dharmacon Inc.; Thermo Scientific, Pittsburgh, PA) using the nucleofector kit (VPA-1003 Amaxa, Lonza, Cologne, GE) and cultured in HEMA for 3 days to assess expansion potential and response to SCF.

### Statistical Analysis

Results are presented as Mean (±SEM) or Mean (±SD) of at least three separate experiments in triplicate. Statistical analysis was performed by Anova (Origin 6.0, Microcal Software, Inc., Northampton, MA), Kruskal–Wallis or Wilcoxon–Mann–Whitney test (two-sided, *p* = 0.05) (SAS software v9.2, SAS Institute, Cary, NC).

## Results

### SCF Elicits Greater Proliferation Responses in PV and CB Than in AB

**Figure 1:**
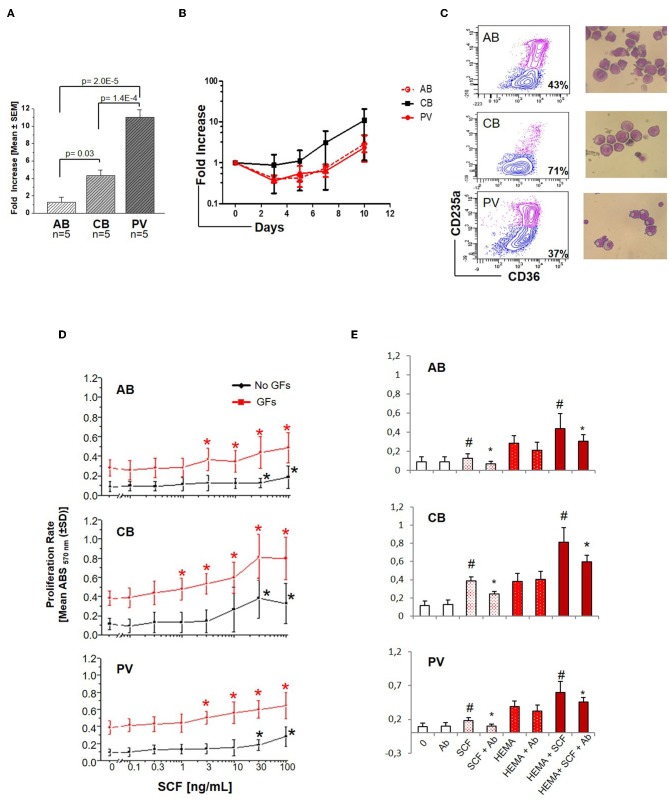
Erythroid cells expanded from PV and CB respond readily to SCF. **(A)** Fold increase of erythroid cells generated by Day-13 in HEMA by AB, CB and PV [Mean (±SEM) of five different donors per group]. Statistical analysis was performed by Anova. **(B)** Fold increase, with respect to Day-0, over time in HEMA of AB, CB, or PV [Mean (±SEM) of three different donors per group]. **(C)** Representative flow cytometry (CD36/CD235a) and morphological (May–Grunwald staining) analyses of representative Day-10 cells which were subjected to phosphoproteomic analyses. CD36/CD235a expression classifies the cultured cells into erythroid progenitors (blue contour) and erythroblasts (purple contour) (33). The frequency of erythroid progenitors from each source is indicated within the quadrant. **(D)** SCF concentration/proliferation response curves of erythroid cells from AB, CB, and PV, as indicated. Cells were GFD and incubated either with increasing concentration of SCF alone (black curves) or in combination with IL-3, EPO, and Dex (growth factors, GFs, red curves) [Mean (±SD) of absorbance_570nm_ (ABS_570nm_) in three separate experiments, each one with a different donor, performed in triplicate]. Statistically different (Kruskall-Wallis Test, *p* < 0.05) with respect to cells cultured with no GFs (black *) or GFs alone (red *). **(E)** Effect of a cKIT neutralizing antibody (Ab) on the proliferation of erythroid cells obtained from AB, CB, and PV induced by SCF (30 ng/mL) either alone (white bars) or in combination with GFs (HEMA, red bars). ^#^Statistically different with respect to 0 or HEMA. *Statistically different with respect to SCF without Ab.

Experiments conducted with 5 donors per source showed that under HEMA conditions (SCF, IL-3, EPO, and Dex) human CD34^+^ cells from PV and CB generate greater numbers of erythroblasts than those from AB by Day-13 (Figure 1A), as previously reported (32, 34–36). However, until Day-10 HEMA of the three sources have similar cell density (fold increase = 1–8 for all sources) (Figure 1B) with a frequency of erythroid progenitors (CD36^+^CD235a^−^ cells) similar in PV and AB (43–37%) and slightly higher in CB (71%) cultures (Figure 1C). To investigate whether the greater expansions observed in cultures of PV and CB underscored an intrinsically greater response to growth stimuli, we compared the proliferation rates of Day-10 erythroid progenitors from different sources upon stimulation with growth factors (GFs) used in HEMA (Figures 1D,E). Erythroid progenitors from the three sources expressed similar proliferation rates in response to GFs (~ 0.4 absorbance, ABS_570nm_). By contrast, the same cells had different proliferation rates in response to SCF (Figure 1D). As originally demonstrated for murine progenitor cells (34), the growth curves of erythroid progenitors from adult sources (AB and PV) remained exponential up to the highest concentration investigated (100 ng/mL) while those of CB plateaued at 30 ng/mL. Furthermore, when used alone, SCF significantly increased proliferation of erythroid progenitors from all sources at 30 ng/mL while in cultures stimulated with GFs, SCF significantly increased proliferation from 1 ng/mL for CB, and 3 ng/mL for AB and PV. Both when used alone and when used with GFs, the effects of SCF (30 ng/mL) on the three sources were neutralized by cKIT-antibodies (Figure 1E). These results indicate that, under the conditions tested, SCF stimulation saturates cKIT signaling only for CB.

### Pathway Analysis Reveals Activation of Canonical c-KIT Signaling in CB and of Non-canonical cKIT Signaling in PV

**Figure 2:**
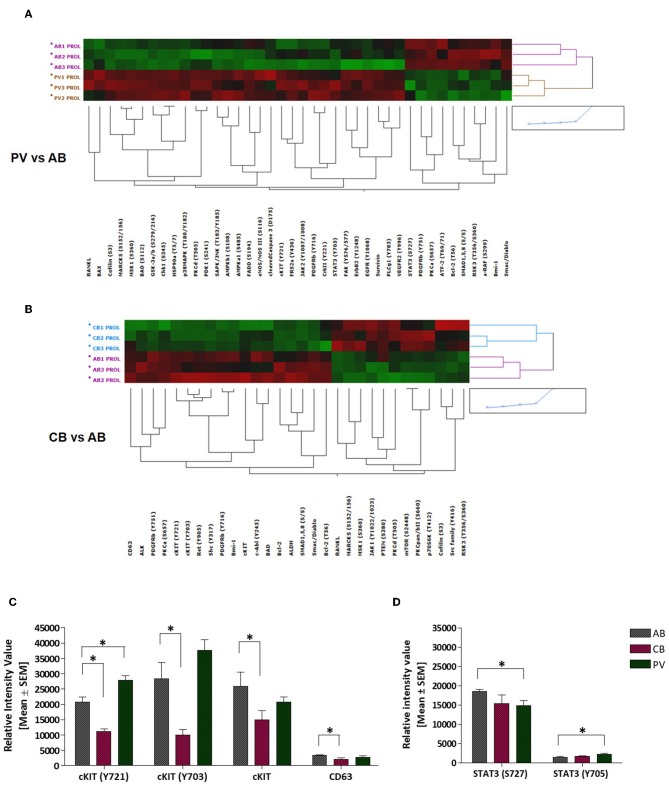
PV and CB express a unique phosphoproteomic profiling suggestive of hyper-activation of non-canonical and canonical cKIT signaling, respectively. **(A,B)** Heatmaps comparing RPPA analysis of protein lysates of erythroid cells from three AB with those from three PV or from three CB (each line a different donor). Quantification of the results is presented in Table S1. **(C)** Quantification of cKITY721, cKITY703, total cKIT, and CD63 content revealed by RPPA in erythroid cells from AB, CB, and PV. Results are expressed as Mean (±SEM) of those observed with three data sets per experimental point. Values statistically different (*p* < 0.05 by Wilcoxon test) are indicated by * (see Table S1 for further details). **(D)** Quantification of STAT3S727 and STAT3Y705 revealed by RPPA in erythroid cells from AB, CB, and PV. Results are expressed as Mean (±SEM) of those observed with three donors per experimental point. Statistically different results (*p* < 0.05 by Wilcoxon test) are indicated by *.

To understand the biochemical basis of different responses to SCF of PV, CB, and AB erythroid progenitors, the cells presented in Figures 1A,B were analyzed by Reverse Phase Protein Array (RPPA). RPPA identified numerous proteins differentially activated between PV (40 proteins) and CB (30 proteins) when compared to AB (Figures 2A,B, Table S1) (two-way hierarchical clustering of all the RPPA data is presented in Figure S1A). Pathway analyses of significant hits predicted that PV and CB differ from AB in the activation state of proteins involved in adhesion/integrin, apoptosis/autophagy, growth factor receptors, stem cell-like properties, cell cycle and TGF-β signaling (Figure S1B, Table S2). Numerous elements downstream to cKIT were differentially activated in CB and PV with respect to AB (differences between CB and PV vs. AB in cKIT signaling are presented in Table 1 while all the different hits are summarized in Table S1). PV showed the greatest number of cKIT elements differentially activated with respect to AB (Figure 2A, Tables S1, S3). In addition to greater activation of JAK2Y1007/1008 and of its downstream partner STAT3Y705 predicted by the presence of JAK2V617F (37), PV expressed levels of cKITY703 and cKITY721, and of their downstream MAPK (pMARCKS, MSK1, AMPKα1 and β1, and p38 MAPK) and PI-3K (eNOS/NOSIII, PDK1, and PKCδ) signaling (38) greater than AB (Figures 2A–D, Tables S1, S3). However, PV expressed lower levels of STAT3S727 (Figure 2D), a site phosphorylated by EPO through ERK/MEK which increases the transactivation potential of the protein (39), indicating that in PV this signaling pathway is less active than in AB. PV showed also activated PLCγ1Y783 but no activation of Src-Y416, the first element of cKIT signaling (40, 41). CB showed lower levels of cKIT, cKITY703 and cKITY721 than AB cells (Figures 2B,C, Table 1). With the exception of ShcY317, PTEN and PKCα which were less activated than in AB, CB showed greater phosphorylation of canonical cKIT signaling proteins (Src; MAPK: MARCKS and MSK1; PI-3K: PKCδ, mTOR, p70S6K; and panPKC/βII) than AB (Figure 2B, Table 1, Tables S1, S3). They also showed greater JAK1 activation than AB. Therefore, in apparent contrast with comparable levels of cKIT activation, overall the signaling profile of CB predicted a stronger activation of canonical cKIT signaling than in AB. This apparent discrepancy was reconciled by the observation that levels of CD63, a tetraspanin superfamily protein (42) that binds cKITY721 accelerating its internalization/degradation (43), were lower in CB than in AB. As cKIT transduces its signal mostly on the plasma membrane (44, 45), we hypothesize that in CB lower levels of CD63 allow cKIT to elicit stronger signals by delaying internalization.

**Table 1:** List of endpoints belonging to the cKIT pathway after the comparison of erythroid cells obtained from CB and PV and those obtained from AB.

**Proteins**	**Cord Blood vs. Adult Blood**		**Polycythemia Vera vs. Adult Blood**	
	**FC**	**Prob > ChiSq**	**FC**	**Prob > ChiSq**
**cKIT**				
cKIT	0.5781	0.0495	0.8007	0.5127
cKIT (Y703)	0.3525	0.0495	1.3222	0.1266
cKIT (Y721)	0.5312	0.0495	1.3381	0.0495
**Y568-dependent MAPK pathway**				
a-RAF (S299)	0.7521	0.2752	0.4094	0.0495
Shc (Y317)	0.4889	0.0495	1.4066	0.1266
Src family (Y416)	1.8656	0.0495	1.0765	0.8273
JAK2 (Y1007/1008)	0.9860	0.8273	1.0679	0.0463
STAT3 (Y705)	1.0978	0.8273	1.4759	0.0495
**Y703-dependent MAPK pathway**				
AMPKα1 (S485)	0.9891	0.8273	1.5255	0.0495
AMPKβ1 (S108)	1.1706	0.2752	1.4603	0.0495
MARCKS (S152/156)	2.5902	0.0495	2.1193	0.0495
MSK1 (S360)	1.4171	0.0495	1.5624	0.0495
p38 MAPK (T180/Y182)	1.9890	0.2752	5.6832	0.0495
PTEN (S380)	1.3690	0.0495	1.3806	0.2752
SAPK/JNK (T183/Y185)	1.6632	0.5127	1.9516	0.0495
STAT3 (S727)	0.8279	0.5127	0.8031	0.0495
**Y721-dependent PI3K pathway**				
eNOS/NOS III (S116)	1.6747	0.1266	1.7393	0.0495
mTOR (S2448)	1.6223	0.0495	1.2063	0.1266
p70 S6K (T412)	2.5314	0.0495	1.6621	0.1266
PDK1 (S241)	1.1951	0.2752	1.4408	0.0495
PKCα (S657)	0.5503	0.0495	0.4540	0.0495
PKCδ (T505)	1.2954	0.0495	1.3079	0.0495
PKCpan/βII (S660)	1.7180	0.0495	1.2922	0.1266
CD63	0.5984	0.0495	0.8000	0.2752
**Y730-dependent PLCγ** **pathway**				
PLCγ1 (Y783)	0.8172	0.2752	1.2433	0.0495
**Y900-dependent SAR pathway**				
CrkII (Y221)	0.7857	0.2752	1.8568	0.0495

*The table shows fold change (FC) values of CB and PV over AB and the relative p-values of the comparison analysis for each single endpoint. FC>2 are shown in red, FC < 0.5 are shown in green; p < 0.05 (Wilcoxon test) are shown in yellow*.

### Biochemical Validation of cKIT Signaling Events Identified by RPPA

**Figure 3:**
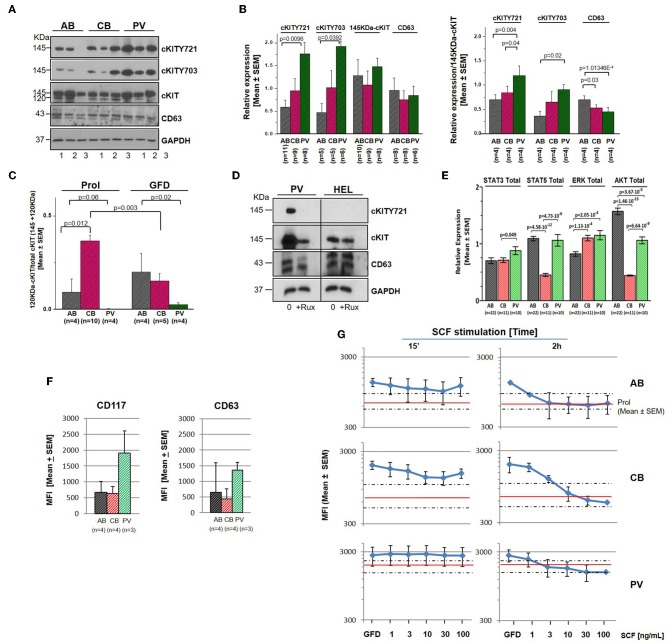
Erythroid cells from PV express greater levels of cKIT721 and cKIT703 than CB and AB. **(A)** WB for cKITY721, cKITY703, total cKIT, CD63, and GAPDH (loading control) of cell lysates from three AB, CB, and PV. **(B)** Quantification of cKITY721, cKITY703, 145KDa-cKIT, and CD63 obtained by WB [Mean (±SEM) of relative intensities with respect to GAPDH (left) and as stoichiometry with respect to 145KDa-cKIT (right)]. **(C)** Quantification of levels of immature 120KDa-cKIT with respect to total (120 + 145 KDa) cKIT obtained by WB. Erythroid cells were analyzed either untreated or after 4 h of GFD. **(D)** WB for CD63, cKIT, and cKITY721 of erythroid cells from one PV and HEL cells, as control, cultured for 48 h with and without ruxolitinib (10 μM). **(E)** Quantification of the total levels of STAT3, STAT5, ERK1/2, and AKT detected by WB in erythroid cells from multiple AB, CB, and PV. In **(A–E)**, number of donors analyzed is indicated. Statistically significant *p*-values were calculated by Anova. **(F)** Cell-surface levels of cKIT and CD63 in erythroid progenitors from multiple donors [Mean (±SEM) Mean Fluorescence Intensity (MFI)]. **(G)** Cell-surface levels of cKIT erythroid progenitors from AB, CB, and PV subjected to GFD and then exposed to increasing concentrations of SCF for 15′ or 2 h [Mean(±SEM) MFI with three separate AB, CB, and PV. Red lines and areas within dotted lines indicate baseline cKIT expression on cells before GFD].

Genome-wide association studies (GWAS) have identified several SNPs lying close to a prominent DNase hypersensitive region ~115 kb upstream of *cKIT* which are associated with variability in red blood cell (RBC) counts, mean RBC volume and mean RBC hemoglobin content observed in the normal population (46, 47). To take into account possible effects of donor genetic makeup and protein metabolism, selected RPPA results were validated by Western Blot (WB) on larger numbers of donors (6–22 donors/sources) (Figure 3). Erythroid cells expanded from this cohort of donors having a presumably heterogeneous genetic makeup showed large differences in cKIT content and metabolism. Although by WB the 145KDa-cKIT content of erythroid cells from PV was not statistically different from that of CB and/or AB (Figures 3A,B), both PV (undetectable) and CB (three-fold greater) contained levels of 120KDa-cKIT significantly different from that of AB (Figure 3C). In addition, WB confirmed that PV contain greater levels of cKITY721 and cKITY703, both in absolute and stoichiometry values, than AB (Figures 3A,B), suggesting that JAK2V617F pre-activates cKIT. This hypothesis was tested by determining that total cKIT and cKITY721 content in PV were greatly reduced by 24h culture with the pan-JAK inhibitor Ruxolitinib (Figure 3D, Figure S3). By WB with large numbers of donors, CB and AB contained similar levels of cKITY721 and cKITY703, both as absolute and stoichiometry values, indicating that differences in levels observed by RPPA were indeed due to a donor sampling effect. This analyses, however, confirmed that by cKIT stoichiometry, CB contained significantly lower CD63 than AB and revealed significantly lower CD63 stoichiometry in PV as well (Figure 3B).

By contrast with cKIT, the low SEMs indicate that the content of STAT3, STAT5, ERK, and AKT was remarkably similar among cells from multiple donors (Figure 3E). PV contained levels of STAT3 and ERK (greater) and AKT (lower) significantly different from AB. ERK content was greater and AKT content lower than AB also in CB which contained also less STAT5 than AB.

### In PV, Altered cKIT Metabolism Includes Longer Cell-Surface Retention Upon Ligand Engagement

**Figure 4:**
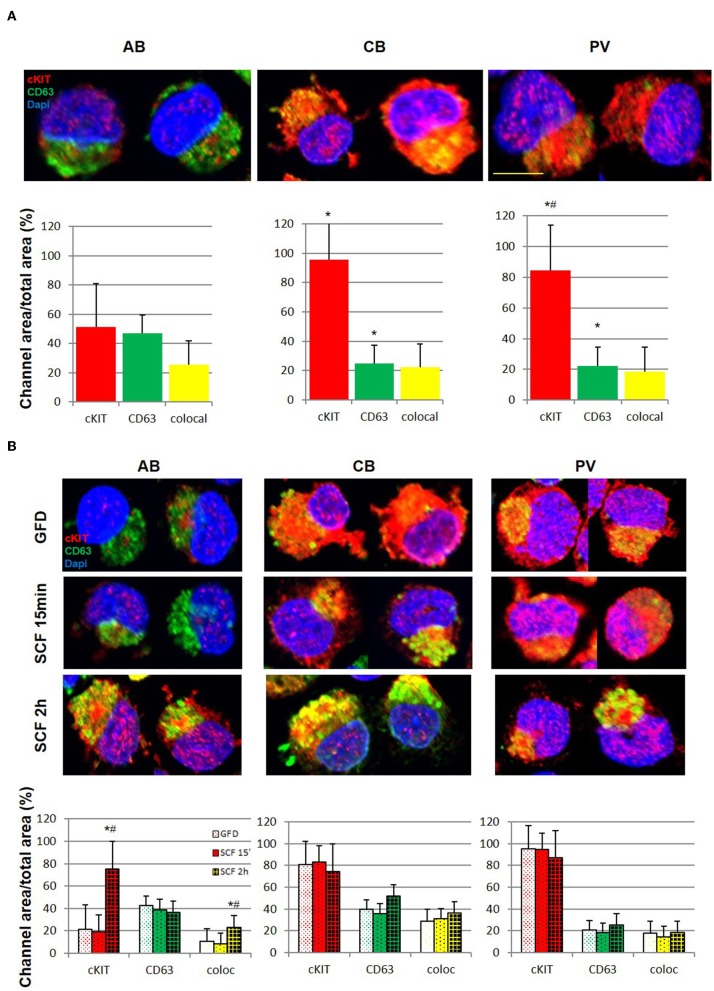
Erythroid progenitors from PV undergo lower cKIT endocytosis upon SCF engagement than those from AB and CB. **(A)** Confocal microscopy with anti-cKIT (red) and anti-CD63 (green) antibodies of two representative permeabilized erythroid cells from AB, CB, and PV. Co-localization of cKIT and CD63 signals are in yellow. Nuclei were counterstained with Dapi (blue). Erythroid cells were recognized by their size (>30 μm) (29). The scale bar represents 20 μm. Results are representative of those obtained in two separate AB, CB, and PV experiments, each one with a different donor. The bar graphs on the bottom indicate the mean (±SEM) fluorescent signals observed in the individual channels in at least 15 erythroid cells per experimental point (a total of >30 cells per bar). Statistically significant (*p* < 0.001 by Anova) differences from AB cells are indicated by *. **(B)** Confocal microscopy with anti-cKIT (red) and anti-CD63 (green) antibodies of two representative permeabilized erythroid cells from AB, CB, and PV subjected to GFD and then stimulation with SCF (100 ng/mL) for 15′ and 2 h. Co-localization of cKIT and CD63 signals are depicted in yellow. Nuclei were counterstained with Dapi (blue). Results are representative of those obtained in two separate AB, CB, and PV experiments, each one with a different donor. The bar graphs on the bottom indicate the Mean (±SEM) fluorescent signals observed in the individual channels in at least 15 erythroid cells per experimental point. Results statistically different (*p* < 0.05 by Anova) with respect to the corresponding GFD or SCF 15′ are indicated by * or #, respectively.

After ligand engagement, 145KDa-cKIT undergoes down-modulation and internalization by endocytosis, the speed of this process determining the extent of the signal delivered by SCF (Figure S2). Since in hematopoietic cell lines the speed of cKIT endocytosis is accelerated by binding of cKITY721 to CD63, we investigated cKIT metabolism in primary erythroid cells. By flow cytometry, similar robust levels of cKIT and CD63 were detected on the surface of erythroid cells from all the sources (Figure 3F, Figure S4). There was a trend toward greater levels of cKIT expression in PV erythroid progenitors which however, given the high variability of cKIT expression in the human population, did not reach statistical significance. Levels of cKIT endocytosis were inferred by determining cell-surface cKIT/SCF concentration curves in erythroid cells subjected to GFD and then exposed to increasing SCF concentrations (0–100 ng/mL, 15′ and 2 h) (Figure 3G). The treatments exerted cell source-specific effects. GFD had no effects on cell-surface expression of cKIT in PV while increased that of AB (two-fold) and CB (three-fold). The magnitude of cKIT up-regulation induced by GFD mirrors the reservoir of immature 120KDa-cKIT which was detected by WB (Figure 3C). In PV, SCF did not affect cell-surface cKIT expression by 15′ and induced modest concentration/dependent down-modulations by 2 h (Figure 3G), suggesting that upon SCF engagement cKIT underwent limited endocytosis and degradation. This long cell-surface retention may underlay the strong cKIT signaling elicited by SCF in PV identified by RPPA. By contrast, in AB and CB cKIT cell-surface expression was down-modulated by SCF in concentration and time-dependent fashions. In AB, down-modulation was modest (two-fold at 30 ng/mL) by 15′ and down to baseline by 2 h (all concentrations), indicating that AB retains activated cKIT on the cell-surface for <15′, in agreement with the limited signaling detected by RPPA. In CB, SCF-induced cKIT down-modulation was partial by 15′ and complete by 2 h only at 30–100 ng/mL, indicating that in CB SCF engagement induces less cKIT endocytosis than in AB, consistently with the robust cKIT signaling upon SCF-stimulation observed by RPPA. These results mirror the SCF concentration/proliferation curves in Figure 1D, further indicating that under the condition of our study SCF stimulation saturates cKIT signaling only in CB. cKIT endocytosis was also inferred by determining cytoplasmic levels of cKIT in permeabilized erythroid cells by confocal microscopy. Cells were analyzed at steady state and following GFD and SCF-stimulation (100 ng/mL, 15′-2 h). These analyses showed that the cytoplasm of PV and CB contain significant more cKIT and less CD63 than AB (Figure 4A). GFD did not affect the cytoplasmic content of cKIT in PV, but reduced by two-fold that in AB (from 50 to 20%) and CB (from 98 to 80%) (Figure 4A vs. Figure 4B). SCF exposure had no effect on the intracellular levels of cKIT in PV and CB but significantly increased that of AB by 2 h (Figure 4B), mirroring the changes in cell-surface expression of cKIT induced by GFD and SCF in the different sources observed by flow-cytometry (Figure 3G). SCF increased the co-localization of CD63 and cKIT in AB only, suggesting that it induces physical CD63/cKIT association mainly in AB. These results indicate that in human erythroid cells activated cKIT undergoes endocytosis at ontogenic- (delayed in CB) and disease- (limited in PV) specific rates.

### SCF Activates Transient and Sustained Canonical cKIT Signaling in AB and CB, Respectively, and Non-canonical cKIT Signaling in PV

**Figure 5:**
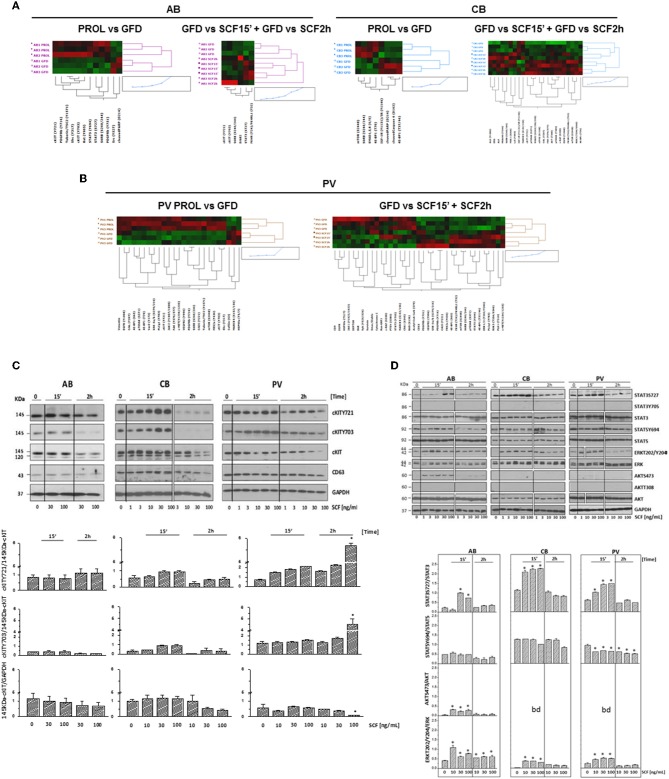
Phosphoproteomic landscaping reveals that PV and CB activate more signals in response to SCF than AB. **(A,B)** Comparison of RPPA from AB, CB, and PV with (GFD) and without GFD (PROL) and among GFD cells with and without exposure to SCF (30 ng/mL) for 15′ and 2 h (three donors/group with the exception of 15′/2 h SCF PV that contain two donors/group, significant hits for each source are indicated in Tables S4–S6). **(C)** WB for cKITY721, cKITY703, cKIT, CD63, and GAPDH (loading control) in erythroid cells from representative AB, CB, and PV. Cells were GFD for 4 h (0) and then exposed to increasing SCF concentration for 15′ or 2 h. Bar graphs on the bottom present the stoichiometry of cKITY721/145KDa-cKIT and cKITY703/145KDa-cKIT and the quantifications of 145KDa-cKIT with respect to GAPDH (three separate experiments, each one with a different donor). *Indicates significant (*p* < 0.001 by Anova) differences from GFD cells. **(D)** WB analyses of STAT3S727, STAT3Y705, STAT5Y694, ERK1/2T202/Y204, and AKTS473 and of the corresponding total proteins from AB, CB, and PV exposed to GFD and then to increasing SCF concentration for 15′ and 2 h. STAT3Y705 and AKTT308 were not detected by WB. Results are representative of those observed in three separate experiments per source. Stoichiometry of STAT3S727, STAT5Y694, and ERK1/2T202/Y204 with respect to the corresponding total proteins is presented on the bottom. Results are representative of those obtained in three separate experiments each one with a different donor. bd, below detection. Values significantly (*p* < 0.001 by Anova) different from GFD are indicated by *. In **(C,D)**, the vertical white lines indicate the conjuncture of results obtained in different Western blots.

cKIT signaling in erythroid cells from different sources was directly assessed by RPPA profiling of PV, CB, and AB subjected to GFD and exposure to SCF (30 ng/mL, 15′-2 h). Heat-maps were created by comparing untreated cells with GFD-cells and GFD-cells with cells exposed for 15′ and 2 h to SCF (Figures 5A,B). GFD significantly altered 25 proteins (22 repressed and three activated) in PV, 12 proteins (10 repressed and two activated) in AB and eight proteins (4 repressed and 4 activated) in CB. Moreover, SCF altered 37 proteins in PV (19 activated and 18 repressed), 23 proteins in CB (20 activated and three repressed) and six in AB (all activated) (Figures 5A,B, Tables S4–S6). Pathway analyses predicted that, with few exceptions, changes induced by GFD and SCF were source-specific (Figure S5, Tables S7–S9). In PV, GFD repressed several receptors (cKIT, EGFR, cMET, PDGFR-β, and VEGFR2). Alterations on cKIT included decreased levels of cKITY703, cKITY721 and of its downstream signaling elements JAK2Y1007/1008, MAPKs, mTOR and PLCγ1Y783 and of the PLCγ1Y783 target Vav3Y173 (48) (Table S4). SCF barely restored the activation of the same proteins.

In PV, SCF did not induce further increase of cKITY703 and cKITY721, both as total and stoichiometry values, while these forms were significantly increased as total levels by SCF in AB and CB (Figures 5A,B, Figures S6A,B, Tables S4–S6), providing further support for the hypothesis that in PV the presence of JAK2V617F prevents GFD to reset cKIT activity. In spite of undetectable SCF-induced cKIT phosphorylation, in PV SCF activated a strong non-canonical signaling which included mTOR and the mTORC1 target S6 Ribosomal Protein 6 (RPS6S240/244), decreased DEPTOR, a component of the mTOR complex mTORC2 (49, 50), and increased PLCγ1Y783 and ERK1/2T202/Y204, when 15′ and 2h data were analyzed in combination (Figure 5B, Table S4).

In CB, SCF robustly activated elements downstream to PI-3K, such as mTOR and its mTORC1 targets RPS6S240/244, that GFD had decreased, and eIF4GS108, p70 S6KT389, and S371, and AKTT308 but did not reduce DEPTOR, indicating that SCF activates a canonical cKIT signaling (Figure 5A, Table S5). Since PI-3K and CD63 both bind Y721, this strong PI-3K activation suggests that CD63 is bound weakly to Y721 in CB.

In AB, SCF did not induced detectable mTOR activation but increased the levels of its target RPS6S240/244, suggesting that in these cells mTOR was activated for <15′ (Figure 5A, Table S6). Only in AB, SCF significantly increased the EPO-R target STAT3S727 (Figure S6C), confirming data obtained in mice (21, 23, 51) and suggesting that in adult erythroid cells cKIT elicits a weaker and more stringently EPO-R-dependent signaling than in fetal cells. Changes in cKITY703 and cKITY721 and in some of their targets were validated by WB (Figures 5C,D). Preliminary experiments indicated that GFD does not alter 120KDa-cKIT content, which remained barely detectable, in PV and in AB but greatly reduced that of CB (Figure 3C). SCF increased by 2 h both total and stoichiometry levels of cKITY703 and cKITY721 in PV at 100 ng/mL, a concentration 3-times greater than that investigated by RPPA. However, probably for the complex cKIT metabolism after engagement, SCF did not induce significant stoichiometry changes in cKITY703 and cKITY721 detectable by WB in AB and CB (Figure S6B). WB provided stoichiometry validation for the increased levels of STAT3S727 and ERK1/2T202/Y204 induced by SCF that were identified by RPPA but did not reach statistical significance for all samples (Figure 5D). By WB, SCF did not activate AKTS473 in CB and PV, validating the RPPA indication that the AKTS473 upstream signaling mTORC2 is not active, and it reduced STAT5Y694 at the stoichiometry levels in PV. Differences observed in pathway activation among sources were validated by measuring cell viability after treatment with 95 FDA-approved inhibitors against the targets included in the RPPA (Figure S7). The results indicated that PV is more sensitive than AB not only to JAK inhibitors but also to inhibitors of cKIT and MEK, a kinase upstream to ERK1/2. Furthermore, as predicted by its strong canonical cKIT signaling, CB was more sensitive than AB to both cKIT and mTOR/PI-3K inhibitors.

### cKIT and CD63 Are Constitutively Associated in PV

**Figure 6:**
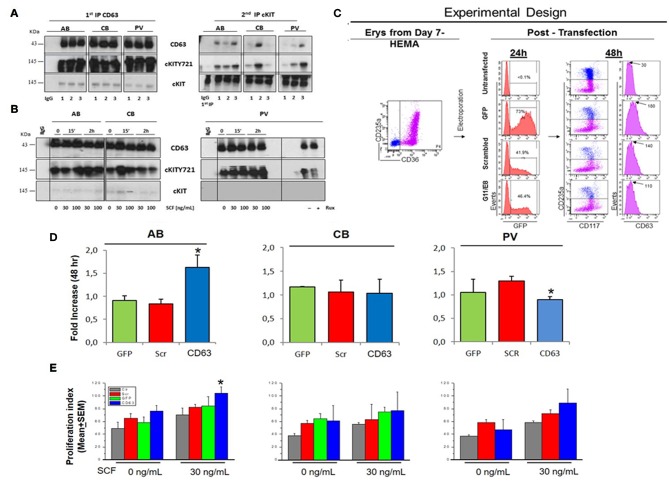
In PV, CD63 does neither associate with cKIT nor restrict proliferation in response to SCF. **(A)** WB for CD63, cKITY721, and cKIT of IPs with CD63 antibodies and with IgG (negative control) of Day-10 cells from three AB, CB, and PV. Second IPs with anti-cKIT antibodies are presented on the right. Similar results were observed with ≥eight donors/group. **(B)** WB with CD63, cKITY721 and cKIT antibodies of IPs obtained with CD63 antibodies and IgG of Day-10 cells expanded from representative AB, CB, and PV. Cells were GFD (0) and then exposed to SCF (30 and 100 ng/mL) for 15′ or 2 h. Cells from PV were also cultures for 48 h with and without Ruxolitinib. **(C)** Experimental design of CD63 suppression by shRNA. Flow-cytometry for CD36-CD235a of the Day-7 CB cells before being transfected with *GFP, GFP*-tagged scrambled-shRNA or *GFP*-tagged-*CD63* shRNA (two shRNAs, G11 and G8) vectors. Transfection efficiency was evaluated 24h later based on GFP expression. Efficiency of CD63 suppression was evaluated 48 h later based on levels of CD63 expression on the surface of CD235a^neg^cKIT^pos^ erythroid progenitors. **(D)** Fold Increase after 48 h culture with SCF of AB, CB and PV transfected with GFP, scrambled or CD63-shRNA. *Significantly different (by Anova) from GFP and scr. **(E)** Proliferation of AB, CB and PV untransfected (Ctr), transfected with GFP, scrambled-shRNA or CD63-shRNA cultured for 48 h with and without SCF. *Statistically different (*p* < 0.05 by Anova) from untransfected. In **(D,E)**, results are presented as Mean(±SEM) of three separate AB, CB, and PV experiments. In **(B)**, the vertical white line indicates the conjuncture of results obtained in different Western blots.

To investigate the role of CD63 in determining the speed of cKIT endocytoses and therefore, the variegation of its signaling, the physical association between CD63 and cKIT in cells from AB, CB, and PV was investigated by IPs (Figures 6A,B). At steady state, CD63-antibodies pulled-down high levels of CD63 and cKITY721 and barely detectable levels of 145KDa-cKIT, from all the three sources (Figure 6A). Second IPs with cKIT-antibodies pulled-down robust levels of 145KDa-cKIT low levels of both cKITY721 and CD63 in stoichiometric amounts. These results indicate that in erythroid cells CD63 binds cKIT phosphorylated at Y721. In SCF-treated cells, CD63-IPs normalized for protein content pulled-down similar levels of CD63 from all sources (Figure 6B). In PV, the level of cKITY721 pulled-down were not affected by SCF but greatly reduced by Ruxolitinib (Figure 6B, Figure S3). By contrast, SCF greatly increased the amount of cKITY721 pulled-down from AB (30 ng/mL, 2 h and 100 ng/mL, 15′-2 h) but induced modest increases (30 ng/mL, 15′ only) or even decreased (100 ng/mL, 2 h) cKITY721 pull-down in CB. Since CD63/cKITY721 association results in endocytosis, these results are consistent with the confocal microscopy observations indicating that SCF increased cytoplasmic co-localization of cKIT/CD63 mainly in AB (Figure 4B). Biological consequences of CD63/cKIT association in different sources were assessed by loss-of-function studies with shRNA (Figures 6C–E). Control experiments confirmed that only CD63-shRNA reduced CD63 expression in erythroid and HEL cells (Figure 6C, Figure S8). CD63-shRNA-treated cells from PV proliferated significantly less (by 20%) while those from adult blood proliferated 2-times more than the corresponding GFP-/scrambled-shRNA-treated cells. CD63-shRNA did not affect CB proliferation (Figure 6D). Lastly, CD63-shRNA did not affect proliferation in response to SCF (30ng/mL) in PV and CB while significantly increased that of AB (Figure 6E). These results indicate that the strength of association with CD63 regulates surface retention of activated cKIT in erythroid cells from different sources. In PV, *JAK2*V617F interferes with the endocytosis signaling of CD63 retaining the CD63/cKIT complex on the cell surface, while in CB the lower levels of CD63 expression delay endocytosis of the receptor.

## Discussion

**Figure 7:**
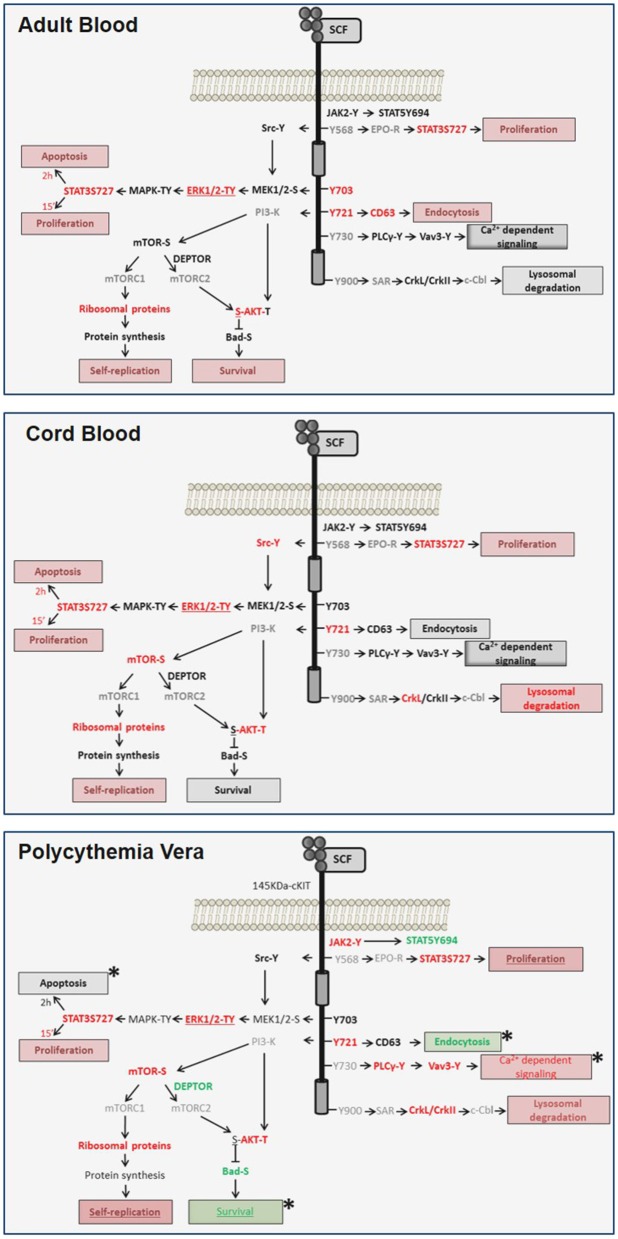
Diagram of the transient and sustained canonical cKIT signaling activated by SCF, respectively, in AB (**top panel**) and CB (**middle panel**) and of the non-canonical signaling activated in PV (**bottom panel**) identified in this study. Events found unchanged, upregulated or down-regulated are in black, red and green fonts, respectively. Events not included in the RPPA array are in gray. Expected biological end-points are summarized in boxes and their level of activation is color-coded as the events. Differences between PV and AB are indicated by *.

Signaling architecture mapping identified in PV a strong signature predictive of levels of cKIT activation qualitatively and quantitatively greater than in AB. The PV*JAK2*V617F-activated cKIT remained responsive to SCF but activated a non-canonical signaling characterized by brief activation of MAPK-signaling, robust activation of the mTORC1-branch downstream to PI-3K/mTOR and PLCγ1/Vav3 but undetectable Src activation and inhibition of mTORC2 (Figure 7). Evidence is provided that this non-canonical activation is probably determined by abnormalities in cKIT metabolism induced by JAK2V617F and although it was detailed in erythroid cells, this non-canonical signaling may be relevant for all hematopoietic stem/progenitor cells from PV. This hypothesis is based on the recognition that the biological consequences of the ERK1/2 and mTOR abnormalities observed in PV predict increased self-replication that is an exquisite property of hematopoietic stem cells. Furthermore, it has been recently reported that JAK2 activation, complemented by cKIT signaling, triggers extensive self-renewal of adult multipotent progenitor cells in mice (52).

Screening of FDA-approved inhibitors and shRNA experiments targeting events identified by RPPA validated the biological relevance of abnormalities identified in PV and provided insights on possible druggable targets for the disease. In fact, survival of PV was more sensitive than AB not only to JAK/STAT inhibitors but also to inhibitors of cKIT and MEK (which is upstream of ERK1/2) and of CD63 suggesting that further endeavors to treat the disease should be focused either on MEK inhibitors or CD63 antibodies (53). It may in fact hypothesized that, by neutralizing CD63, these antibodies may restore cKIT metabolism in PV suppressing the non-canonical signaling observed in these cells. Consistently with this conclusion, it was recently reported that MEK inhibition rescues the phenotype of mouse models of myeloproliferative neoplasms (MPN) (54). It would be important, in future studies, to analyze the cKIT signaling and metabolism also in erythroid cells expanded from primary myelofibrosis and essential thrombocytopenia in order to evaluate whether these drugs may be effective also in additional myeloproliferative neoplasms.

PI-3K signaling was poorly active in *JAK2*V617F-PV. Similar results were previously reported in blood mononuclear cells [by WB, (55)], neutrophils [by phosphoproteomic (56)] and CD34^+^ cells [by transcriptosome profiling (56)]. Therefore, it is not surprising that the survival of PV was not more sensitive than AB to inhibitors of mTOR/PI-3K, including rapamycin a derivative of which, Everolimus, is under clinical investigation in MPN (53, 55, 57), predicting that treatments with PI-3K inhibitors alone may have limited efficacy in PV.

Erythroid cells from CB were analyzed to unveil possible ontogenetic-specific differences in cKIT metabolism/signaling because, by contrast with adult cells, respond readily to SCF (34). The comparison of the signaling/metabolism of this receptor in fetal and adult cells identified that in response to SCF both AB and CB activated a canonical cKIT signaling which was transient in AB and prolonged in CB. Analysis of cKIT metabolism identified the critical role played by CD63 in favoring endocytosis of cKIT, and therefore in quenching its signaling, in adult cells. In spite of the well-described effects of SCF on erythropoiesis, clinical attempts to use this growth factor for the treatment of EPO-unresponsive anemias have not been encouraging so far and an international trial in Diamond Blackfan Anemia was prematurely interrupted by the National Institute of Health after the death of one patient (58). Our observations suggest that CD63 antibodies, by neutralizing CD63, may prolong the canonical signaling of cKIT at physiological SCF levels increasing the expansion of the adult erythroid pool and. therefore, may be useful for the treatment of these anemias because they would not trigger the severe side observed with treating the patients with supra-physiological levels of SCF which also activate other cell lineages, in primis mast cells, responsible for the toxicity of the treatment.

The great variability observed in levels of cKIT expressed by erythroid cells from different donors probably related to their genetic makeup highlights the clinical importance of further studies on the genetic control of cKIT expression in erythropoiesis. We foresee that these studies may elucidate the variegation of disease severity expressed by patients with PV and other congenital or acquired erythroid disorders and be instrumental to design personalized therapies.

## Data Availability Statement

The datasets generated for this study are available at http://capmm.gmu.edu/data.

## Author Contributions

GF and LV designed research, performed experiments, collected and assembled data, performed data analysis and interpretation. FM and MF performed experiments and analyzed data. OP analyzed data. FF and PC performed experiments. GG provided buffy coats discarded from regular blood and cord blood donations and assured compliance of the study with institutional IRB. MM revised the manuscript. AT provided buffy coats from Polycythemia Vera and assured compliance of the study with institutional IRB. EP wrote the manuscript and supervised the phosphoproteomic analyses. AZ and AM designed research, analyzed and interpreted the data, wrote the manuscript.

### Conflict of Interest

EP is a co-inventor of the RPPA and has issued patents on the technology. These patents have been licensed and EP can receive royalties on these licenses. The remaining authors declare that the research was conducted in the absence of any commercial or financial relationships that could be construed as a potential conflict of interest.
