# Supplementary material for: Phosphoproteomic Landscaping Identifies Non-canonical cKIT Signaling in Polycythemia Vera Erythroid Progenitors
Source: Front Oncol. 2019 Nov 22;9:1245. doi: 10.3389/fonc.2019.01245 (PMC6883719; doi:10.3389/fonc.2019.01245)
Supplement: Supplementary file 1 [file Table_1.DOCX]

**Table S1.** **Significant endpoints between erythroid cells obtained from CB and PV and those obtained from AB.** List of significant differences obtained after the comparison of CB and PV erythroid cells with AB erythroid cells. The table shows fold change (FC) values of CB and PV over AB and the relative p values of the two comparison analysis for each single endpoint. FC>2 are shown in red, FC<0.5 are shown in green; p values<0.05 (Wilcoxon test) are shown in yellow.

| **Proteins** | **Cord Blood *vs* Adult Blood** | | **Polycythemia Vera vs Adult Blood** | |
| --- | --- | --- | --- | --- |
|  | **FC** | **Prob>ChiSq** | **FC** | **Prob>ChiSq** |
| **ALDH** | 0.2175 | 0.0495 | 0.9672 | 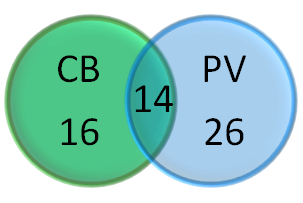0.8273 |
| **ALK** | 0.4278 | 0.0495 | 0.7204 | 0.2752 |
| **AMPKα1 (S485)** | 0.9891 | 0.8273 | 1.5255 | 0.0495 |
| **AMPKβ1 (S108)** | 1.1706 | 0.2752 | 1.4603 | 0.0495 |
| **a-RAF (S299)** | 0.7521 | 0.2752 | 0.4094 | 0.0495 |
| **ATF-2 (T69/71)** | 0.4556 | 0.1266 | 0.0488 | 0.0495 |
| **BAD** | 0.6938 | 0.0495 | 1.0208 | 0.8273 |
| **BAD (S112)** | 1.1388 | 0.5127 | 1.4262 | 0.0495 |
| **BAX** | 0.8947 | 0.5127 | 1.3866 | 0.0495 |
| **Bcl-2** | 0.3052 | 0.0495 | 0.6247 | 0.1266 |
| **Bcl-2 (T56)** | 0.8162 | 0.0495 | 0.8693 | 0.0495 |
| **Bmi-1** | 0.4136 | 0.0495 | 0.4698 | 0.0495 |
| **c-Abl (Y245)** | 0.3061 | 0.0495 | 0.8308 | 0.5127 |
| **CD63** | 0.5984 | 0.0495 | 0.8000 | 0.2752 |
| **Chk1 (S345)** | / | / | 10.1819 | 0.0463 |
| **cKIT** | 0.5781 | 0.0495 | 0.8007 | 0.5127 |
| **cKIT (Y703)** | 0.3525 | 0.0495 | 1.3222 | 0.1266 |
| **cKIT (Y721)** | 0.5312 | 0.0495 | 1.3381 | 0.0495 |
| **cleaved Caspase 3 (D175)** | 1.0975 | 0.5127 | 1.9920 | 0.0495 |
| **Cofilin (S3)** | 3.8508 | 0.0495 | 3.3970 | 0.0495 |
| **CrkII (Y221)** | 0.7857 | 0.2752 | 1.8568 | 0.0495 |
| **EGFR (Y1068)** | 0.6904 | 0.5127 | 1.4437 | 0.0495 |
| **eNOS/NOS III (S116)** | 1.6747 | 0.1266 | 1.7393 | 0.0495 |
| **ErbB2 (Y1248)** | 1.0079 | 0.8273 | 1.1361 | 0.0495 |
| **FADD (S194)** | 1.0135 | 0.8273 | 1.4284 | 0.0495 |
| **FAK (Y576/577)** | 0.7867 | 0.1266 | 1.1461 | 0.0495 |
| **FRS2α (Y436)** | 0.8578 | 0.5127 | 1.6232 | 0.0495 |
| **GSK-3α/β (S279/216)** | 0.8689 | 0.1266 | 1.3909 | 0.0495 |
| **HSP90a (T5/7)** | 0.9457 | 0.5127 | 1.5994 | 0.0495 |
| **JAK1 (Y1022/1023)** | 1.7188 | 0.0495 | 1.4401 | 0.1266 |
| **JAK2 (Y1007/1008)** | 0.9860 | 0.8273 | 1.0679 | 0.0463 |
| **MARCKS (S152/156)** | 2.5902 | 0.0495 | 2.1193 | 0.0495 |
| **MSK1 (S360)** | 1.4171 | 0.0495 | 1.5624 | 0.0495 |
| **mTOR (S2448)** | 1.6223 | 0.0495 | 1.2063 | 0.1266 |
| **p38 MAPK (T180/Y182)** | 1.9890 | 0.2752 | 5.6832 | 0.0495 |
| **p70 S6K (T412)** | 2.5314 | 0.0495 | 1.6621 | 0.1266 |
| **PDGFRβ (Y716)** | 0.4275 | 0.0495 | 1.7030 | 0.0495 |
| **PDGFRβ (Y751)** | 0.6946 | 0.0495 | 0.7198 | 0.0495 |
| **PDK1 (S241)** | 1.1951 | 0.2752 | 1.4408 | 0.0495 |
| **PKCα (S657)** | 0.5503 | 0.0495 | 0.4540 | 0.0495 |
| **PKCδ (T505)** | 1.2954 | 0.0495 | 1.3079 | 0.0495 |
| **PKCpan/βII (S660)** | 1.7180 | 0.0495 | 1.2922 | 0.1266 |
| **PLCγ1 (Y783)** | 0.8172 | 0.2752 | 1.2433 | 0.0495 |
| **PTEN (S380)** | 1.3690 | 0.0495 | 1.3806 | 0.2752 |
| **RANKL** | 2.0373 | 0.0495 | 1.5574 | 0.0495 |
| **Ret (Y905)** | 0.3709 | 0.0495 | 1.3554 | 0.1266 |
| **RSK3 (T356/S360)** | 1.6660 | 0.0495 | 0.2671 | 0.0495 |
| **SAPK/JNK (T183/Y185)** | 1.6632 | 0.5127 | 1.9516 | 0.0495 |
| **Shc (Y317)** | 0.4889 | 0.0495 | 1.4066 | 0.1266 |
| **Smac/Diablo** | 0.7550 | 0.0495 | 0.8907 | 0.0495 |
| **SMAD1 (S/S)/SMAD5 (S/S)/SMAD8 (S/S)** | 0.6627 | 0.0495 | 0.5846 | 0.0495 |
| **Src family (Y416)** | 1.8656 | 0.0495 | 1.0765 | 0.8273 |
| **STAT3 (S727)** | 0.8279 | 0.5127 | 0.8031 | 0.0495 |
| **STAT3 (Y705)** | 1.0978 | 0.8273 | 1.4759 | 0.0495 |
| **Survivin** | 0.9981 | 0.8273 | 1.1541 | 0.0463 |
| **VEGFR2 (Y996)** | 0.8576 | 0.2752 | 1.2760 | 0.0495 |
