# Supplementary material for: Phosphoproteomic Landscaping Identifies Non-canonical cKIT Signaling in Polycythemia Vera Erythroid Progenitors
Source: Front Oncol. 2019 Nov 22;9:1245. doi: 10.3389/fonc.2019.01245 (PMC6883719; doi:10.3389/fonc.2019.01245)
Supplement: Supplementary file 2 [file Table_2.DOCX]

**Table S2. Pathway analyses of the differences observed by RPPA between CB and PV, and AB.** Red and green indicate events activated (A) or suppressed (I) with respect to AB. To be noted that activation/suppression of events that suppress/activate individual pathways exerts negative (-)/positive (+) effects of the pathways. Common differences are highlighted in yellow.

| **Pathway** | **CB *vs* AB** | | **PV *vs* AB** | |
| --- | --- | --- | --- | --- |
|  | **Protein** | **Events** | **Protein** | **Events** |
| **Adhesion/Integrin Signaling** | Cofilin (S3) (I) | (n= -2, +1) | Cofilin (S3) (I) | (n= -1, +2) |
|  | c-Abl (Y245) (A) |  | CrkII (Y221) (I) |  |
|  | Src family (Y416) (A) |  | FAK (Y576/577) (A) |  |
| **AKT Proliferation Signaling** | PTEN (S380) (I) | (n= +1) | AMPKα1 (S485) (A) | (n= -2, +3) |
|  |  |  | AMPKβ1 (S108) (A) |  |
|  |  |  | eNOS/NOS III (S116) (I) |  |
|  |  |  | GSK-3α/β (S279/216) (A) |  |
|  |  |  | PDK1 (S241) (A) |  |
| **Apoptosis/Autophagy** | Bcl-2 (T56) (A) | (n= -2, +2) | Bcl-2 (T56) (A) | (n= -3, +4) |
|  | Smac/Diablo (A) |  | Smac/Diablo (A) |  |
|  | BAD (A) |  | BAD (S112) (I) |  |
|  | Bcl-2 |  | BAX (A) |  |
|  |  |  | cleaved Caspase 3 (D175) (A) |  |
|  |  |  | FADD (S194) (A) |  |
|  |  |  | Survivin (I) |  |
| **Growth Factor Receptors** | cKIT (Y721) (A) | (n= -6) | cKIT (Y721) (A) | (n= -1, +5) |
|  | PDGFRβ (Y716) (A) |  | PDGFRβ (Y716) (A) |  |
|  | PDGFRβ (Y751) (A) |  | PDGFRβ (Y751) (A) |  |
|  | ALK |  | EGFR (Y1068) (A) |  |
|  | cKIT (A) |  | ErbB2 (Y1248) (A) |  |
|  | cKIT (Y703) (A) |  | VEGFR2 (Y996) (A) |  |
| **JAK/STAT Signaling** | JAK1 (Y1022/1023) (A) | (n= +1) | JAK2 (Y1007/1008) (A) | (n= -1, +2) |
|  |  |  | STAT3 (S727) (A) |  |
|  |  |  | STAT3 (Y705) (A) |  |
| **MAPKs Proliferation Signaling** | MARCKS (S152/156) (A) | (n= -2, +5) | MARCKS (S152/156) (A) | (n= -4, +6) |
|  | MSK1 (S360) (A) |  | MSK1 (S360) (A) |  |
|  | PKCα (S657) (A) |  | PKCα (S657) (A) |  |
|  | PKCδ (T505) (A) |  | PKCδ (T505) (A) |  |
|  | RSK3 (T356/S360) (A) |  | RSK3 (T356/S360) (A) |  |
|  | PKC pan/βII (S660) (A) |  | a-RAF (S299) (A) |  |
|  | Shc (Y317) (A) |  | ATF-2 (T69/71) (A) |  |
|  |  |  | FRS2α (Y436) (A) |  |
|  |  |  | p38 MAPK (T180/Y182) (A) |  |
|  |  |  | PLCγ1 (Y783) (A) |  |
| **Non-Canonical Signaling** | RANKL (A) | (n= -1, +1) | RANKL (A) | (n= +1) |
|  | CD63 (A) |  |  |  |
| **Stemness** | Bmi-1 (A) | (n= -2) | Bmi-1 (A) | (n= -1) |
|  | ALDH (A) |  |  |  |
| **TGFβ signaling** | SMAD1 (S/S)/SMAD5 (S/S)/SMAD8 (S/S) (A) | (n= -1) | SMAD1 (S/S)/SMAD5 (S/S)/SMAD8 (S/S) (A) | (n= -1) |
| **mTOR Proliferation Signaling** | mTOR (S2448) (A) | (n= +2) | / | / |
|  | p70 S6K (T412) (A) |  |  |  |
| **Thyroid hormone Signaling (enucleation)** | Ret (Y905) (A) | (n= -1) | / | / |
| **Cell Cycle Control** | / | / | Chk1 (S345) (A) | (n= +1) |
| **Stress Signaling** | / | / | HSP90α (T5/7) (A) | (n= +2) |
|  |  |  | SAPK/JNK (T183/Y185) (A) |  |
