# Supplementary material for: Phosphoproteomic Landscaping Identifies Non-canonical cKIT Signaling in Polycythemia Vera Erythroid Progenitors
Source: Front Oncol. 2019 Nov 22;9:1245. doi: 10.3389/fonc.2019.01245 (PMC6883719; doi:10.3389/fonc.2019.01245)
Supplement: Supplementary file 3 [file Table_3.DOCX]

**Table S3. Phosphoproteomic landscaping reveals numerous differences in the activation state of proteins involved in cKIT signaling among erythroid cells from CB and PV and those from AB.** The association of individual signaling events with specific cKIT phosphorylation sites is described by indicating endpoints downstream of the different cKIT phosphorylation sites in CB, PV or both compared to AB. In green are endpoints less expressed in CB/PV compared to AB. In red are endpoints more expressed in CB/PV than AB. Some of these events are induced also by EPO-R (37,60).

| **only in CB** | **both in CB and PV** | **only in PV** |
| --- | --- | --- |
| cKIT (Y721) |  | cKIT (Y721) |
| cKIT (Y703) |  |  |
| cKIT |  |  |
| CD63 |  |  |
| **Y567-dependent MAPK pathway** | | |
| Shc (Y317) |  | a-RAF (S299) |
| Src family (Y416) |  |  |
| **Y703-dependent MAPK pathway** | | |
| PTEN (S380) | MARCKS (S152/156) | AMPKα1 (S485) |
|  | MSK1 (S360) | AMPKβ1 (S108) |
|  |  | p38 MAPK (T180/Y182) |
|  |  | SAPK/JNK (T183/Y185) |
|  |  | STAT3 (S727) |
| **Y719-dependent PI3K pathway** | | |
| mTOR (S2448) | PKCα (S657) | eNOS/NOS III (S116) |
| p70 S6K (T412) | PKCδ (T505) | PDK1 (S241) |
| PKC pan/βII (S660) |  |  |
| **Y728-dependent PLCγ pathway** | | |
|  |  | PLCγ1 (Y783) |
| **Hemopoietic cytokine superfamily signaling** | | |
| JAK1 (Y1022/1023) |  | JAK2 (Y1007/1008) |
|  |  | STAT3 (Y705) |
