# Supplementary material for: Phosphoproteomic Landscaping Identifies Non-canonical cKIT Signaling in Polycythemia Vera Erythroid Progenitors
Source: Front Oncol. 2019 Nov 22;9:1245. doi: 10.3389/fonc.2019.01245 (PMC6883719; doi:10.3389/fonc.2019.01245)
Supplement: Supplementary file 4 [file Table_4.DOCX]

**Table S4. Statistical analyses of events induced by GFD and SCF stimulation in PV.** List of significant differences obtained after the comparison of PROL and SCF-treated PV with GFD PV. The table shows fold change (FC) values of PROL, 15min SCF, 2h SCF and 15min+2h SCF over GFD PV and the relative p values of the four comparison analysis for each single endpoint. FC>2 are shown in red, FC<0.5 are shown in green; p values<0.05 (Wilcoxon test) are shown in yellow, 0.05<p value<0.1 are shown in blue.

| **ANALYZED PROTEINS** | **PROL *vs* GFD** | | **SCF *vs* GFD** | | | | | |
| --- | --- | --- | --- | --- | --- | --- | --- | --- |
|  | **PROL** | **Prob>ChiSq** | **15 min** | **Prob>ChiSq** | **2h** | **Prob>ChiSq** | **15min + 2h** | **Prob>ChiSq** |
| **4E-BP1 (S65)** | 2.2051 | 0.0495 | 2.2390 | 0.0833 | 2.7663 | 0.0833 | 2.5026 | 0.0339 |
| **4E-BP1 (T37/46)** | 1.2180 | 0.2752 | 1.1629 | 0.0833 | 1.2945 | 0.0833 | 1.2287 | 0.0339 |
| **4E-BP1 (T70)** | 1.6113 | 0.0495 | 1.0124 | 0.5637 | 1.5124 | 0.5637 | 1.2624 | 0.4795 |
| **Acetyl-CoA Carboxylase (S79)** | 0.9924 | 0.8273 | 0.9412 | 0.2482 | 0.8863 | 0.0833 | 0.9137 | 0.0771 |
| **ATF-2 (T69/71)** | 0.1260 | 0.3758 | 1.2842 | 0.5637 | 0.3310 | 0.5637 | 0.8076 | 1.0000 |
| **BAD (S136)** | 0.9211 | 0.2752 | 0.9248 | 0.0833 | 0.8552 | 0.0833 | 0.8900 | 0.0339 |
| **BAK** | 1.0060 | 0.8273 | 0.8197 | 0.2482 | 0.8026 | 0.0833 | 0.8111 | 0.0771 |
| **Bcl-2** | 0.1930 | 0.2752 | 0.1702 | 0.2482 | 0.1681 | 0.2482 | 0.1692 | 0.1573 |
| **CD63** | 0.9560 | 0.8273 | 1.2511 | 0.0833 | 1.3562 | 0.0833 | 1.3037 | 0.0339 |
| **CD9** | 1.0559 | 0.1266 | 0.9568 | 0.0833 | 0.9402 | 0.0833 | 0.9485 | 0.0323 |
| **Chk1 (S345)** | 0.8802 | 0.1266 | 0.6822 | 0.0833 | 0.6849 | 0.0833 | 0.6836 | 0.0339 |
| **cKIT (Y703)** | 1.5495 | 0.0495 | 1.1254 | 0.5637 | 1.4650 | 0.2482 | 1.2952 | 0.2888 |
| **cKIT (Y721)** | 1.3383 | 0.0495 | 1.0361 | 1.0000 | 1.2984 | 0.5637 | 1.1673 | 0.7237 |
| **cleaved Caspase 6 (D162)** | 0.6586 | 0.5127 | 0.4797 | 0.2482 | 0.5060 | 0.2482 | 0.4928 | 0.1573 |
| **Cofilin (S3)** | 0.4624 | 0.0495 | 1.1618 | 0.5637 | 1.2658 | 0.2482 | 1.2138 | 0.2888 |
| **c-RAF (S338)** | 0.9321 | 0.2752 | 1.0257 | 1.0000 | 0.9293 | 0.0833 | 0.9775 | 0.2888 |
| **CrkII (Y221)** | 1.4223 | 0.0495 | 1.0372 | 0.5637 | 1.2265 | 0.0833 | 1.1319 | 0.1573 |
| **CrkL (Y207)** | 1.3004 | 0.0495 | 1.2353 | 0.2482 | 1.3567 | 0.2482 | 1.2960 | 0.1573 |
| **Cytochrome C** | 0.9196 | 0.5127 | 0.9424 | 1.0000 | 0.9333 | 0.0833 | 0.9379 | 0.2888 |
| **DEPTOR** | 0.9662 | 0.5127 | 0.7568 | 0.0833 | 0.8371 | 0.0833 | 0.7969 | 0.0339 |
| **EGFR** | 0.9381 | 0.5127 | 0.9345 | 0.2482 | 0.9022 | 0.0833 | 0.9183 | 0.0771 |
| **EGFR (Y1068)** | 1.2533 | 0.0495 | 0.9291 | 0.5637 | 1.0803 | 0.5637 | 1.0047 | 1.0000 |
| **ERK1/2 (T202/Y204)** | 1.1177 | 0.5127 | 1.2507 | 0.0833 | 1.2272 | 0.0833 | 1.2390 | 0.0339 |
| **FAK (Y576/577)** | 1.1454 | 0.0495 | 0.9923 | 0.5637 | 1.0402 | 1.0000 | 1.0163 | 0.7237 |
| **FKHR (T24)/FKHRL1 (T32)** | 1.3587 | 0.1266 | 1.6576 | 0.2482 | 1.9185 | 0.0833 | 1.7881 | 0.0771 |
| **FRS2α (Y436)** | 1.1704 | 0.0495 | 0.9339 | 0.0833 | 1.0752 | 1.0000 | 1.0045 | 0.2888 |
| **GSK-3α/β (S279/216)** | 1.1385 | 0.0463 | 1.0198 | 0.5536 | 1.1442 | 0.0756 | 1.0820 | 0.1536 |
| **HSP90a (T5/7)** | 0.5406 | 0.0495 | 0.4580 | 0.0833 | 0.4564 | 0.0833 | 0.4572 | 0.0339 |
| **JAK1 (Y1022/1023)** | 1.0724 | 0.8273 | 0.8816 | 0.5637 | 0.7812 | 0.0833 | 0.8314 | 0.1573 |
| **JAK2 (Y1007/1008)** | 1.0864 | 0.0463 | 1.0212 | 0.5637 | 1.0671 | 0.5637 | 1.0441 | 0.4795 |
| **MARCKS (S152/156)** | 0.8888 | 0.0495 | 0.9042 | 1.0000 | 0.8421 | 0.0833 | 0.8731 | 0.2888 |
| **Met (Y1234/1235)** | 1.1532 | 0.0495 | 1.0355 | 0.2482 | 1.0562 | 0.0833 | 1.0459 | 0.0771 |
| **mTOR (S2448)** | 1.3199 | 0.0495 | 1.4087 | 0.0833 | 1.7463 | 0.0833 | 1.5775 | 0.0339 |
| **p70 S6K (S371)** | 1.4303 | 0.0495 | 1.0659 | 0.5637 | 1.5490 | 0.0833 | 1.3074 | 0.1573 |
| **PDGFRβ (Y716)** | 1.3983 | 0.0495 | 1.0306 | 0.5637 | 1.3392 | 0.0833 | 1.1849 | 0.1573 |
| **PDGFRβ (Y751)** | 0.9796 | 0.8273 | 1.1396 | 0.0833 | 1.1726 | 0.0833 | 1.1561 | 0.0339 |
| **PLCγ1 (Y783)** | 1.3260 | 0.0495 | 1.0966 | 0.0833 | 1.0987 | 0.0833 | 1.0976 | 0.0339 |
| **PLK1 (T210)** | 0.9987 | 0.8273 | 1.0751 | 0.0833 | 1.0016 | 1.0000 | 1.0383 | 0.2888 |
| **PTEN (S380)** | 0.9804 | 0.5127 | 1.0503 | 0.5637 | 0.8830 | 0.0833 | 0.9666 | 0.4795 |
| **Ras-GRF1** | 1.0244 | 1.0000 | 1.0114 | 0.5637 | 0.9415 | 0.0833 | 0.9764 | 0.4795 |
| **RSK3 (T356/S360)** | 1.5047 | 0.5127 | 4.0803 | 0.0833 | 3.5310 | 0.0833 | 3.8057 | 0.0339 |
| **S6 Ribosomal Protein (S240/244)** | 4.0819 | 0.0495 | 5.3393 | 0.0833 | 11.9436 | 0.0833 | 8.6414 | 0.0339 |
| **Shc (Y317)** | 1.4214 | 0.0495 | 0.9695 | 1.0000 | 1.2751 | 0.2482 | 1.1223 | 0.4795 |
| **Smac/Diablo** | 0.9305 | 0.2752 | 0.9438 | 1.0000 | 0.8721 | 0.0833 | 0.9079 | 0.2888 |
| **STAT3 (Y705)** | 1.1908 | 0.2752 | 0.9673 | 0.2482 | 0.9214 | 0.0833 | 0.9444 | 0.0771 |
| **STAT5 (Y694)** | 1.2621 | 0.2752 | 1.3139 | 0.5637 | 1.8492 | 0.0833 | 1.5815 | 0.1573 |
| **Survivin** | 1.0131 | 0.5002 | 0.9235 | 0.0756 | 0.9381 | 0.5536 | 0.9308 | 0.1498 |
| **Syk (Y525/526)** | 1.0183 | 0.6579 | 0.8754 | 0.0833 | 0.9202 | 0.5637 | 0.8978 | 0.1573 |
| **Tuberin/TSC2 (Y1571)** | 1.0629 | 0.0495 | 1.0204 | 1.0000 | 1.0212 | 1.0000 | 1.0208 | 1.0000 |
| **Vav3 (Y173)** | 1.1014 | 0.0495 | 1.0788 | 0.2482 | 1.0754 | 0.2482 | 1.0771 | 0.1573 |
| **VEGFR2 (Y996)** | 1.2082 | 0.0495 | 1.0172 | 0.5637 | 1.1302 | 0.0833 | 1.0737 | 0.1573 |
| **Vimentin** | 1.0697 | 0.0463 | 0.9512 | 1.0000 | 1.0621 | 0.5637 | 1.0066 | 0.7237 |
