# Supplementary material for: Phosphoproteomic Landscaping Identifies Non-canonical cKIT Signaling in Polycythemia Vera Erythroid Progenitors
Source: Front Oncol. 2019 Nov 22;9:1245. doi: 10.3389/fonc.2019.01245 (PMC6883719; doi:10.3389/fonc.2019.01245)
Supplement: Supplementary file 5 [file Table_5.DOCX]

**Table S5. Statistical analyses of events induced by GFD and SCF stimulation in CB.** List of significant differences obtained after the comparison of PROL and SCF-treated CB with GFD CB. The table shows fold change (FC) values of PROL, 15min SCF, 2h SCF over GFD CB and the relative p values of the three comparison analysis for each single endpoint. FC>2 are shown in red, FC<0.5 are shown in green; p values<0.05 (Wilcoxon test) are shown in yellow.

| **ANALYZED PROTEINS** | **PROL *vs* GFD** | | **SCF *vs* GFD** | | | |
| --- | --- | --- | --- | --- | --- | --- |
|  | **PROL** | **Prob>ChiSq** | **15 min** | **Prob>ChiSq** | **2h** | **Prob>ChiSq** |
| **4E-BP1 (S65)** | 1.1572 | 0.8273 | 2.0864 | 0.2752 | 1.6977 | 0.2752 |
| **4E-BP1 (T37/46)** | 0.7075 | 0.0463 | 0.8000 | 0.2683 | 1.0097 | 0.5002 |
| **4E-BP1 (T70)** | 1.4336 | 0.0495 | 1.0552 | 0.5127 | 1.4234 | 0.0495 |
| **AKT (T308)** | 1.1034 | 0.8273 | 1.7947 | 0.0495 | 1.4514 | 0.5127 |
| **ALK** | 1.2696 | 0.5127 | 1.4526 | 0.0495 | 1.4671 | 0.1266 |
| **ALK (Y1586)** | 1.2552 | 0.5127 | 1.6775 | 0.0495 | 1.6432 | 0.1266 |
| **ATF-2 (T71)** | 0.9475 | 0.8273 | 0.7475 | 0.8273 | 0.4402 | 0.5127 |
| **cKIT (Y721)** | 1.3577 | 0.2752 | 1.5202 | 0.0495 | 1.5588 | 0.1266 |
| **cleaved Caspase 6 (D162)** | 0.2796 | 0.0495 | 1.1670 | 0.8273 | 1.1184 | 0.8273 |
| **cleaved PARP (D214)** | 0.3305 | 0.0495 | 1.0419 | 0.8273 | 0.9576 | 0.8273 |
| **c-RAF (S338)** | 1.2262 | 0.2752 | 1.3684 | 0.0495 | 1.2930 | 0.2752 |
| **CrkL (Y207)** | 1.1916 | 0.5127 | 1.9154 | 0.0495 | 1.4333 | 0.1266 |
| **EGFR (Y1068)** | 1.2403 | 0.5127 | 1.7796 | 0.0495 | 1.7518 | 0.2752 |
| **eIF4G (S1108)** | 1.9317 | 0.2752 | 2.9264 | 0.0495 | 2.3862 | 0.2752 |
| **ERG** | 1.6612 | 0.5127 | 1.7217 | 0.0495 | 1.5754 | 0.2752 |
| **ERK1/2 (T202/Y204)** | 0.4483 | 0.1266 | 0.7422 | 0.8273 | 0.9585 | 0.8273 |
| **FAK (Y576/577)** | 1.0785 | 0.5127 | 1.2599 | 0.2752 | 1.2034 | 0.0495 |
| **FKHR (T24)/FKHRL1 (T32)** | 1.6603 | 0.2752 | 5.0385 | 0.0495 | 3.0547 | 0.0495 |
| **IGF-1R (Y1131)/IR (Y1146)** | 0.8298 | 0.0495 | 0.8474 | 0.0495 | 0.9394 | 0.1266 |
| **Lck (Y505)** | 1.2332 | 0.5127 | 1.4018 | 0.0495 | 1.0929 | 0.5127 |
| **LKB1 (S428)** | 2.1024 | 0.2752 | 1.6690 | 0.2752 | 1.4765 | 0.2752 |
| **MARCKS (S152/156)** | 1.0782 | 0.2752 | 0.8000 | 0.0495 | 0.9792 | 0.8273 |
| **MSK1 (S360)** | 1.2077 | 0.2752 | 1.3187 | 0.0495 | 0.9127 | 0.5127 |
| **mTOR (S2448)** | 1.6738 | 0.0495 | 1.8509 | 0.0495 | 1.8449 | 0.0495 |
| **p70 S6K (S371)** | 1.2281 | 0.2752 | 1.3663 | 0.0495 | 1.6709 | 0.0495 |
| **p70 S6K (T389)** | 0.8534 | 0.3758 | 2.0471 | 0.0495 | 1.9716 | 0.0495 |
| **PRAS40 (T246)** | 1.0849 | 0.2752 | 1.2721 | 0.0495 | 1.1878 | 0.0495 |
| **Ret (Y905)** | 1.5069 | 0.2752 | 1.4453 | 0.2752 | 2.0710 | 0.1266 |
| **RSK3 (T356/S360)** | 1.1596 | 0.5127 | 3.0348 | 0.0495 | 2.9088 | 0.1266 |
| **S6 Ribosomal Protein (S240/244)** | 9.2382 | 0.0495 | 4.1038 | 0.0495 | 6.9554 | 0.0495 |
| **SMAD1 (S/S)/SMAD5 (S/S)/SMAD8 (S/S)** | 1.2879 | 0.0495 | 1.1083 | 0.5127 | 0.8691 | 0.2752 |
| **SMAD2 (S245/250/255)** | 1.2814 | 0.2752 | 1.9372 | 0.0495 | 2.1347 | 0.1266 |
