# Supplementary material for: Phosphoproteomic Landscaping Identifies Non-canonical cKIT Signaling in Polycythemia Vera Erythroid Progenitors
Source: Front Oncol. 2019 Nov 22;9:1245. doi: 10.3389/fonc.2019.01245 (PMC6883719; doi:10.3389/fonc.2019.01245)
Supplement: Supplementary file 6 [file Table_6.DOCX]

**Table S6. Statistical analyses of events induced by GFD and SCF stimulation in AB.** List of significant differences obtained after the comparison of PROL and SCF-treated AB with GFD AB. The table shows fold change (FC) values of PROL, 15min SCF, 2h SCF over GFD AB and the relative p values of the three comparison analysis for each single endpoint. FC>2 are shown in red, FC<0.5 are shown in green; p values<0.05 (Wilcoxon test) are shown in yellow.

| **ANALYZED PROTEINS** | **PROL *vs* GFD** | | **SCF *vs* GFD** | | | |
| --- | --- | --- | --- | --- | --- | --- |
|  | **PROL** | **Prob>ChiSq** | **15 min** | **Prob>ChiSq** | **2h** | **Prob>ChiSq** |
| **cKIT (Y703)** | 1.9862 | 0.0495 | 0.9367 | 0.5127 | 1.4840 | 0.0495 |
| **cKIT (Y721)** | 1.5361 | 0.0495 | 1.0064 | 0.5127 | 1.3662 | 0.0495 |
| **cleaved Caspase 6 (D162)** | 0.2359 | 0.1266 | 0.8974 | 0.5127 | 0.7626 | 0.5127 |
| **cleaved PARP (D214)** | 0.3361 | 0.0495 | 0.8635 | 0.5127 | 0.7738 | 0.5127 |
| **ErbB2** | 1.0685 | 0.8273 | 1.2142 | 0.0495 | 2.7391 | 0.0495 |
| **FKHR (T24)/FKHRL1 (T32)** | 1.3560 | 0.5127 | 3.5621 | 0.0495 | 2.3845 | 0.2752 |
| **LKB1 (S428)** | 2.0778 | 0.2752 | 1.8070 | 0.1266 | 1.6807 | 0.8273 |
| **PDGFRβ (Y716)** | 1.5508 | 0.0495 | 1.0127 | 0.8273 | 1.3278 | 0.2752 |
| **PDGFRβ (Y751)** | 1.1294 | 0.0495 | 0.9999 | 0.8273 | 0.9176 | 0.5127 |
| **Ret (Y905)** | 1.7699 | 0.0495 | 0.9061 | 0.2752 | 1.4363 | 0.5127 |
| **S6 Ribosomal Protein (S240/244)** | 3.6349 | 0.0495 | 2.7249 | 0.2752 | 5.0233 | 0.0495 |
| **Shc (Y317)** | 1.5329 | 0.0495 | 0.9818 | 0.8273 | 1.2118 | 0.5127 |
| **Src (Y527)** | 0.7773 | 0.0495 | 1.0424 | 0.5127 | 1.1858 | 0.8273 |
| **STAT3 (S727)** | 1.1408 | 0.0495 | 1.3278 | 0.0495 | 1.2199 | 0.0495 |
| **STAT5 (Y694)** | 3.5884 | 0.0495 | 1.5610 | 0.5127 | 2.7999 | 0.2752 |
| **Tuberin/TSC2 (Y1571)** | 1.1253 | 0.0495 | 1.0135 | 0.8273 | 1.0319 | 0.2752 |
