# Supplementary material for: Phosphoproteomic Landscaping Identifies Non-canonical cKIT Signaling in Polycythemia Vera Erythroid Progenitors
Source: Front Oncol. 2019 Nov 22;9:1245. doi: 10.3389/fonc.2019.01245 (PMC6883719; doi:10.3389/fonc.2019.01245)
Supplement: Supplementary file 7 [file Table_7.DOCX]

**Table S7. Pathway analyses of events observed in PV exposed to GFD and then stimulated with SCF.** Red and green indicate events activated or suppressed with respect to unmanipulated cells (PROL). To be noted that activation/suppression of events that suppress/activate individual pathways exerts negative (-)/positive (+) effects of the pathways. Common differences are highlighted in yellow.

| **Pathway** | **Prol *vs* GFD** | | **15 min+2h *vs* GFD** | |
| --- | --- | --- | --- | --- |
|  | **Protein** | **Events** | **Protein** | **Events** |
| **Adhesion/Integrin Signaling** | Cofilin (S3) (I) | (n= +6) | / | / |
|  | CrkII (Y221) (I) |  |  |  |
|  | CrkL (Y207) (A) |  |  |  |
|  | FAK (Y576/577) (A) |  |  |  |
|  | Vav3 (Y173) (A) |  |  |  |
|  | Vimentin (A) |  |  |  |
| **AKT Proliferation Signaling** | GSK-3α/β (S279/216) (A) | (n= -1) | / | / |
| **Apoptosis/**  **Autophagy**  **Signaling** | / | / | BAD (S136) (I) | (n= +1) |
| **Growth Factor Receptors** | cKIT (Y703) (A) | (n= +6) | PDGFRβ (Y751) (A) | (n= +1) |
|  | cKIT (Y721) (A) |  |  |  |
|  | EGFR (Y1068) (A) |  |  |  |
|  | Met (Y1234/1235) (A) |  |  |  |
|  | PDGFRβ (Y716) (A) |  |  |  |
|  | VEGFR2 (Y996) (A) |  |  |  |
| **JAK/STAT**  **Signaling** | JAK2 (Y1007/1008) (A) | (n= +1) | / | / |
| **MAPKs Proliferation Signaling** | PLCγ1 (Y783) (A) | (n= -1, +3) | PLCγ1 (Y783) (A) | (n= +3) |
|  | FRS2α (Y436) (A) |  | ERK1/2 (T202/Y204) |  |
|  | MARCKS (S152/156) (A) |  | RSK3 (T356/S360) (A) |  |
|  | Shc (Y317) (A) |  |  |  |
| **Non-Canonical Signalings** | / | / | CD9 | (n= -1, +1) |
|  |  |  | CD63 |  |
| **Stemness** | / | / | / | / |
| **TGFβ**  **Signaling** | / | / | / | / |
| **mTOR Proliferation Signaling** | 4E-BP1 (S65) (I) | (n= +6) | 4E-BP1 (S65) (I) | (n= +5) |
|  | mTOR (S2448) (A) |  | mTOR (S2448) (A) |  |
|  | S6 Ribosomal Protein (S240/244) (A) |  | S6 Ribosomal Protein (S240/244) (A) |  |
|  | 4E-BP1 (T70) (I) |  | 4E-BP1 (T37/46) (I) |  |
|  | p70 S6K (S371) (A) |  | DEPTOR (A) |  |
|  | Tuberin/TSC2 (Y1571) (I) |  |  |  |
| **Thyroid hormone Signaling** | / | / | / | / |
| **Cell Cycle**  **Control** | / | / | Chk1 (S345) (A) | (n= -1) |
| **Stress Signaling** | HSP90α (T5/7) (A) | (n= -1) | HSP90α (T5/7) (A) | (n= -1) |
