# Supplementary material for: Phosphoproteomic Landscaping Identifies Non-canonical cKIT Signaling in Polycythemia Vera Erythroid Progenitors
Source: Front Oncol. 2019 Nov 22;9:1245. doi: 10.3389/fonc.2019.01245 (PMC6883719; doi:10.3389/fonc.2019.01245)
Supplement: Supplementary file 8 [file Table_8.DOCX]

**Table S8. Pathway analyses of events observed in AB exposed to GFD and then stimulated with SCF.** Red and green indicate events activated or suppressed with respect to unmanipulated cells (PROL). To be noted that activation/suppression of events that suppress/activate individual pathways exerts negative (-)/positive (+) effects of the pathways. Common differences are highlighted in yellow.

| **Pathway** | **Prol *vs* GFD** | | **15 min *vs* GFD** | | **2h *vs* GFD** |  |
| --- | --- | --- | --- | --- | --- | --- |
|  | **Protein** | **Events** | **Protein** | **Events** | **Protein** | **Events** |
| **Adhesion/Integrin**  **Signaling** | Src (Y527) (I) | (n= +1) | / | / | / | / |
| **AKT Proliferation**  **Signaling** | / | / | FKHR (T24)/FKHRL1 (T32) (I) | (n= +1) | / | / |
| **Apoptosis/**  **Autophagy**  **Signaling** | cleaved PARP (D214) (I) | (n= -1) | / | / | / | / |
| **Growth Factor**  **Receptors** |  | (n= +4) | ErbB2 | (n= +1) | ErbB2 | (n= +3) |
|  | cKIT (Y721) (A) |  |  |  | cKIT (Y719) (A) |  |
|  | cKIT (Y703) (A) |  |  |  | cKIT (Y703) (A) |  |
|  | PDGFRβ (Y716) (A) |  |  |  |  |  |
|  | PDGFRβ (Y751) (A) |  |  |  |  |  |
| **JAK/STAT**  **Signaling** | STAT3 (S727) (A) | (n= +2) | STAT3 (S727) (A) | (n= +1) | STAT3 (S727) (A) | (n= +1) |
|  | STAT5 (Y694) (A) |  |  |  |  |  |
| **MAPKs Proliferation**  **Signaling** | Shc (Y317) (A) | (n= +1) | / | / | / | / |
| **Non-Canonical**  **Signalings** | / | / | / | / | / | / |
| **Stemness** | / | / | / | / | / | / |
| **TGFβ Signaling** | / | / | / | / | / | / |
| **mTOR Proliferation**  **Signaling** | S6 Ribosomal Protein (S240/244) (A) | (n= +2) | / | / | S6 Ribosomal Protein (S240/244) (A) | (n= +1) |
|  | Tuberin/TSC2 (Y1571) (I) |  |  |  |  |  |
| **Thyroid hormone**  **Signaling** | Ret (Y905) (A) | (n= +1) | / | / | / | / |
| **Cell Cycle**  **Control** | / | / | / | / | / | / |
| **Stress Signaling** | / | / | / | / | / | / |
