# Supplementary material for: Phosphoproteomic Landscaping Identifies Non-canonical cKIT Signaling in Polycythemia Vera Erythroid Progenitors
Source: Front Oncol. 2019 Nov 22;9:1245. doi: 10.3389/fonc.2019.01245 (PMC6883719; doi:10.3389/fonc.2019.01245)
Supplement: Supplementary file 9 [file Table_9.DOCX]

**Table S9. Pathway analyses of events observed in CB exposed to GFD and then stimulated with SCF.** Red and green indicate events activated or suppressed with respect to unmanipulated cells (PROL). To be noted that activation/suppression of events that suppress/activate individual pathways exerts negative (-)/positive (+) effects of the pathways. Common differences are highlighted in yellow.

| **Pathway** | **Prol *vs* GFD** | | **15 min *vs* GFD** | | **2h *vs* GFD** |  |
| --- | --- | --- | --- | --- | --- | --- |
|  | **Protein** | **Events** | **Protein** | **Events** | **Protein** | **Events** |
| **Adhesion/Integrin**  **Signaling** | / | / | CrkL (Y207) (A) | (n= +1) | FAK (Y576/577) (A) | (n= +1) |
| **AKT Proliferation**  **Signaling** | / | / | FKHR (T24)/FKHRL1 (T32) (I) | (n= +3) | FKHR (T24)/FKHRL1 (T32) (I) | (n= +2) |
|  |  |  | PRAS40 (T246) (I) |  | PRAS40 (T246) (I) |  |
|  |  |  | AKT (T308) (A) |  |  |  |
| **Apoptosis/**  **Autophagy Signaling** | cleaved Caspase 6 (D162) (A) | (n= -2) | / | / | / | / |
|  | cleaved PARP (D214) (I) |  |  |  |  |  |
| **Growth Factor**  **Receptors** | IGF-1R (Y1131)/IR (Y1146) (A) | (n= -1) | IGF-1R (Y1131)/IR (Y1146) (A) | (n= -1, +5) | / | / |
|  |  |  | ALK (A) |  |  |  |
|  |  |  | ALK (Y1586) (A) |  |  |  |
|  |  |  | cKIT (Y721) (A) |  |  |  |
|  |  |  | EGFR (Y1068) (A) |  |  |  |
|  |  |  | ERG (A) |  |  |  |
| **JAK/STAT**  **Signaling** | / | / | / | / | / | / |
| **MAPKs Proliferation Signaling** | / | / | c-RAF (S338) (A) | (n= -2, +3) | / | / |
|  |  |  | Lck (Y505) (I) |  |  |  |
|  |  |  | MARCKS (S152/156) (A) |  |  |  |
|  |  |  | MSK1 (S360) (A) |  |  |  |
|  |  |  | RSK3 (T356/S360) (A) |  |  |  |
| **Non-Canonical**  **Signalings** | / | / | / | / | / | / |
| **Stemness** | / | / | / | / | / | / |
| **TGFβ**  **Signaling** | SMAD1 (S/S)/SMAD5 (S/S)/SMAD8 (S/S) (A) | (n= +1) | SMAD2 (S245/250/255) (A) | (n= +1) | / | / |
| **mTOR Proliferation Signaling** | mTOR (S2448) (A) | (n= -1, +3) | mTOR (S2448) (A) | (n= +5) | mTOR (S2448) (A) | (n= +5) |
|  | S6 Ribosomal Protein (S240/244) (A) |  | S6 Ribosomal Protein (S240/244) (A) |  | S6 Ribosomal Protein (S240/244) (A) |  |
|  | 4E-BP1 (T70) (I) |  |  |  | 4E-BP1 (T70) (I) |  |
|  |  |  | p70 S6K (S371) (A) |  | p70 S6K (S371) (A) |  |
|  |  |  | p70 S6K (T389) (A) |  | p70 S6K (T389) (A) |  |
|  | 4E-BP1 (T37/46) (I) |  | eIF4G (S1108) (A) |  |  |  |
| **Thyroid hormone Signaling** | / | / | / | / | / | / |
| **Cell Cycle**  **Control** | / | / | / | / | / | / |
| **Stress Signaling** | / | / | / | / | / | / |
