# Supplementary material for: Phosphoproteomic Landscaping Identifies Non-canonical cKIT Signaling in Polycythemia Vera Erythroid Progenitors
Source: Front Oncol. 2019 Nov 22;9:1245. doi: 10.3389/fonc.2019.01245 (PMC6883719; doi:10.3389/fonc.2019.01245)
Supplement: Supplementary file 10 [file Presentation_1.pdf]

Figure S1. Phosphoproteomic analysis on AB, CB and PV Erythroid cells. A) Two-way hierarchical clustering or erythroid cells derived from CB (blue), AB (purple) and PV (brown) in different conditions: proliferation (PROL, square), growth factor deprivation (GFD, cross), 15 min (circle) and 2h (diamond) treatment with SCF for the expression/activation of 151 total, cleaved and phospho-endpoints. **B)** Pathway analyses of proteins differentially expressed according to their predicted biological state (inhibitory or activatory) in CB and PV with respect to AB. The phosphoprotein hits included in each individual pathway are rather small. The individual hits included in each pathway are listed in Supplementary Table S2.

Figure S2. Diagram of current knowledge on cKIT metabolism after SCF engagement based on experiments published in murine mast cell lines (see text for further detail). Modified from Abella JV, Park M. Breakdown of endocytosis in the oncogenic activation of receptor tyrosine kinases. Am J Physiol Endocrinol Metab. (2009) 296:E973–84. doi: 10.1152/ajpendo.90857.2008

Figure S3. Western blot and IP analyses of PV and HEL cells after Ruxolitinib treatment. A) WB analyses for JAK2Y1007/1008 and STAT5Y694 in JAK2V617-positive HEL cells either untreated or incubated with ruxolitinib [10μM] for 24-48h. JAK2V617-negative K562 cells were analyzed as negative control. GAPDH was analyzed as loading control. **B)** WB analyses for CD63, cKITY721 and cKIT of IPs obtained with antibodies against cKIT and with IgG, as negative control, of lysates from erythroid cells from one PV donor cultured with and without ruxolitininb [10μM] for 48h. Supernatants of the first IP were subjected to a second IP with anti-CD63 antibodies that were probed by WB for CD63, cKIT and cKITY721. HEL cells were analyzed in parallel as negative control.

Figure S4. Representative flow-cytometry analyses for the expression of CD117 (an antibody that recognized cKIT) and CD63 on the surface of Day-10 progentior cells ($CD36^{pos}CD235a^{neg}$, blue contour in the left panel) expanded from AB, CB and PV. Contour analyses indicated that these populations co-expressed cKIT and CD63 at the single cell level (data not shown).

Figure S5. Pathway analyses of changes initiated by GFD and SCF stimulation for 15' and 2h in AB, CB and PV. A) Phosphoproteomic hits differentially expressed in the three sources in the different conditions were divided in individual signaling pathways and classified according to both their expression and their predicted effects (inhibitory or activatory). Individuals hits included in each pathway are summarized in Supplementary Tables S4-S6.

Figure S6. Quantitative representation of the RPPA data presented in Fig. 5A,B. A) Quantification of the differences in cKITY721, cKITY703, total cKIT and CD63 revealed by RPPA in erythroid cells from AB, CB and PV GFD and then stimulated with SCF for 15' and 2h. Results are expressed as Mean (±SEM) of those observed with two-three data sets per experimental point. Values statistically different ($p<0.05$ by Wilcoxon test) are indicated by *.. See also Supplementary Tables S4-S6 for further details. **B)** Stoichiometry of the levels of cKITY721 and cKITY703 with respect to total cKIT observed by RPPA in erythroid progenitors from AB, CB and PV subjected to the various manipulations indicated. See legend of Figure 4A,B for further details. **C)** Quantification of differences in STAT3S727, STAT3Y705, STAT5Y694, ERK1/2T202/Y204 and AKTT308 revealed by RPPA in erythroid cells from AB, CB and PV subjected to the manipulations described in Fig.5A,B.

Figure S7. Survival of erythroid cells from PV and CB is more dependent on cKIT signaling than that of cells from AB. A,B) Viability of erythroid cells from AB, CB and PV cultured for 24h in HEMA supplemented with 95 FDA-approved anti-cancer compounds (each lane one compound) included in the Selleck Chemicals library of signaling inhibitors [all tested at 10µM]. The 95 inhibitors, specified on the bottom, were selected because they targets pathways (summarized on the top) found to be differentially activated among erythroid cells from the three sources by RPPA. Results are expressed as percent of the viability observed in the absence of chemical. * indicates levels of inhibitions >20% greater than those observed with AB.

Figure S8. Forced expression of CD63-shRNA reduces CD63 expressed by HEL cells. A) Bright and fluorescent field observations of HEL cells either untreated or electroporated with an expression vector containing GFP-tagged CD63-shRNA. Magnification 10X. **B)** WB analyses for cKIT and CD63 of HEL cells either untreated or transfected with scrambled-shRNA or CD63-shRNAs (E8 and G11). GADPH was analyzed as loading control. **C)** Flow-cytometry analyses for GFP, CD63 and CD117 (cKIT) of HEL cells either untreated of transfected with scrambled-shRNA or CD63-shRNA. Levels of CD63 and CD117 expressed by GFP-negative and GFP-positive cells are indicated in purple and orange, respectively. GFP-positive cells from the CD63-shRNA group express less CD63 than the corresponding GFP-negative cells and untreated or scrambled-shRNA group. Expression of CD117 is the same in all groups.

A

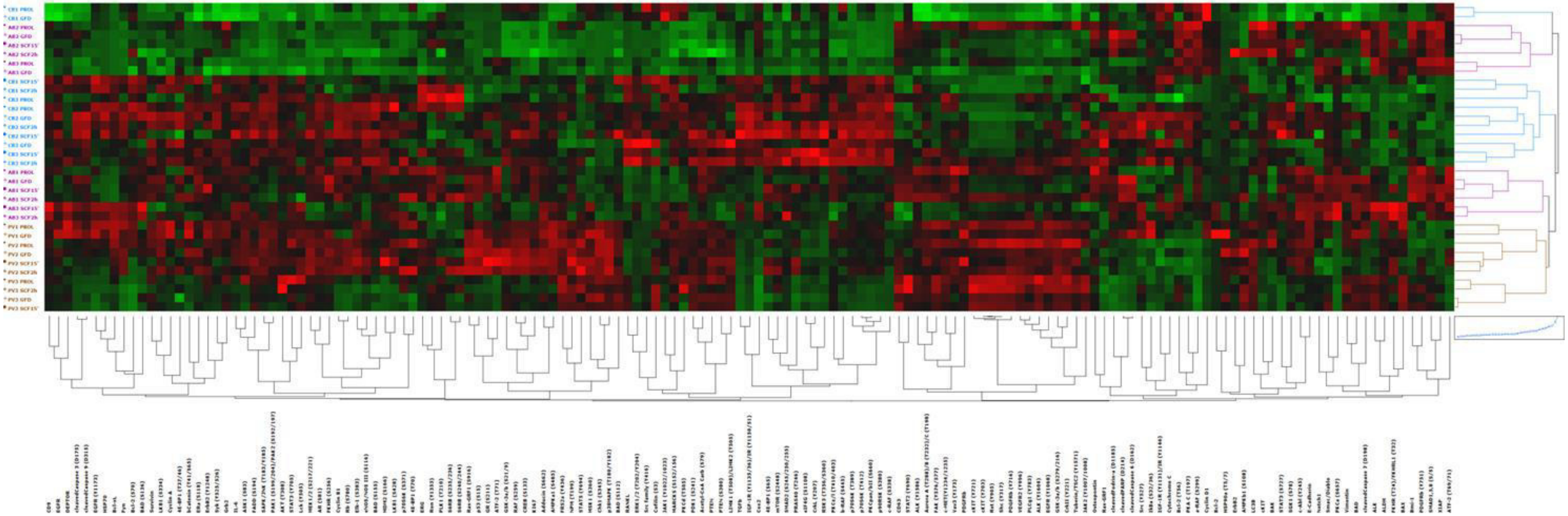

B

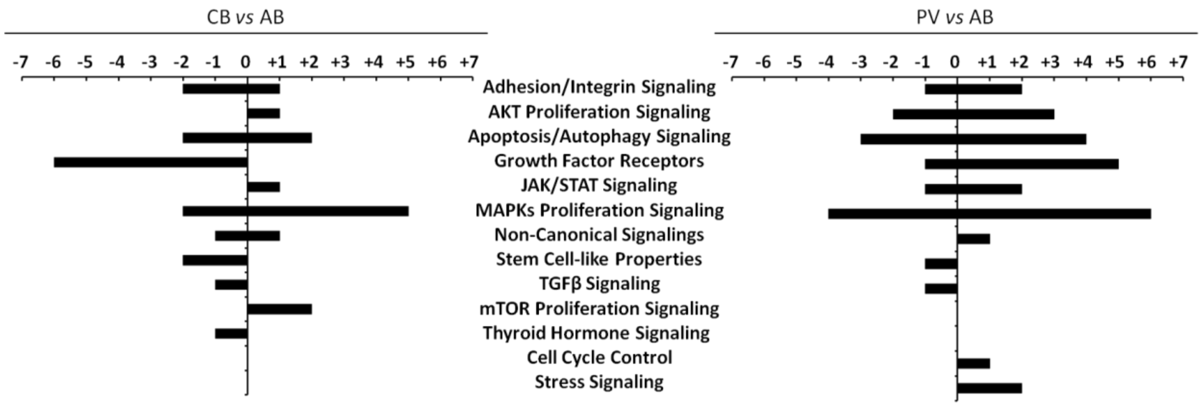

Figure S1A-B

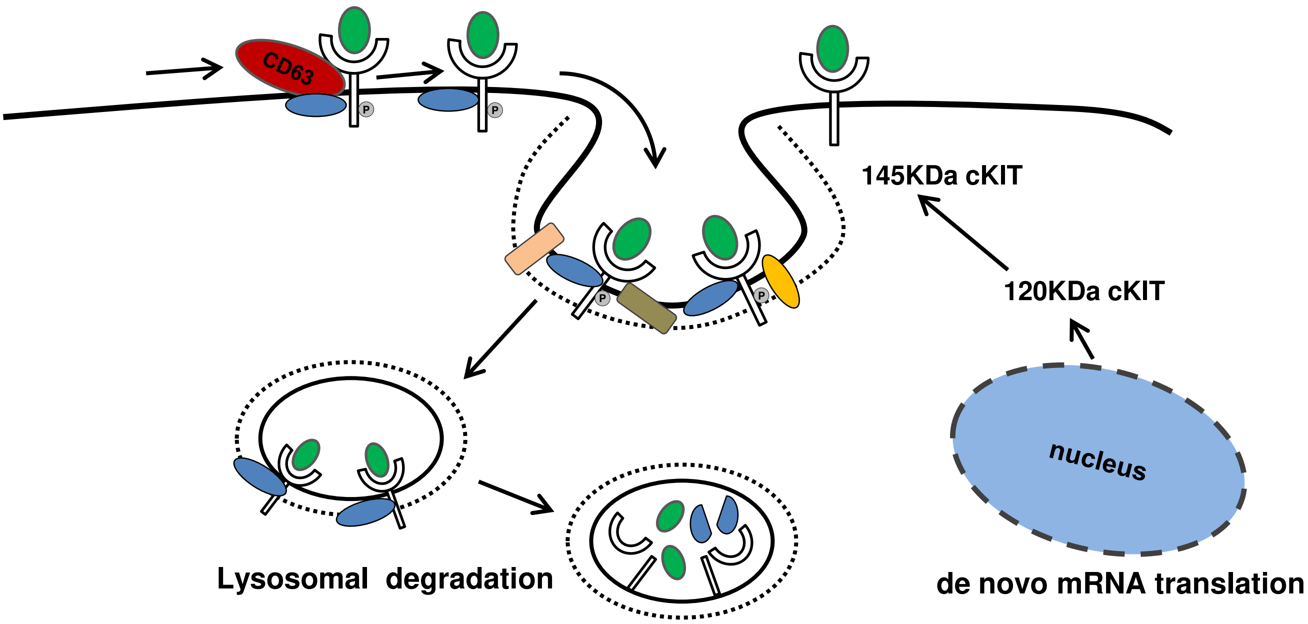
CD63
P
145KDa cKIT
120KDa cKIT
nucleus
Lysosomal degradation
de novo mRNA translation

Figure S2

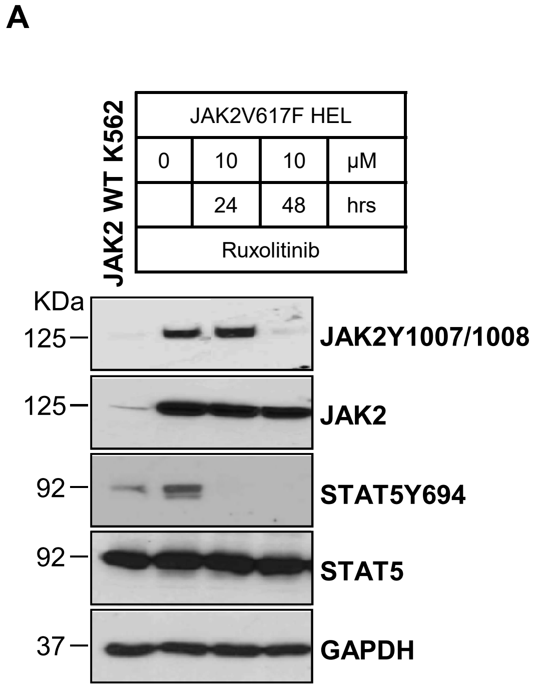
A
JAK2 WT K562
JAK2V617F HEL
0
10
10
μM
24
48
hrs
Ruxolitinib
KDa
125
125
92
92
37
JAK2Y1007/1008
JAK2
STAT5Y694
STAT5
GAPDH

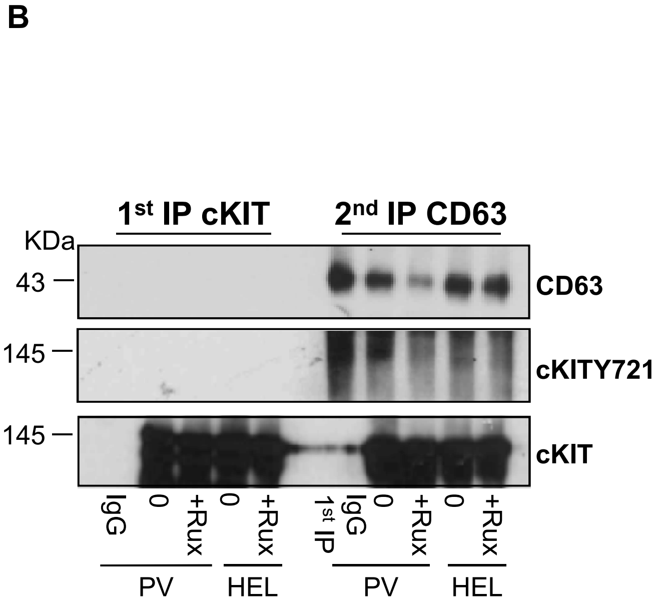
B
1st IP cKIT
2nd IP CD63
KDa
43
145
145
CD63
cKITY721
cKIT
IgG
0
+Rux
0
+Rux
1st IP
IgG
0
+Rux
0
+Rux
PV
HEL
PV
HEL

Figure S3A-B

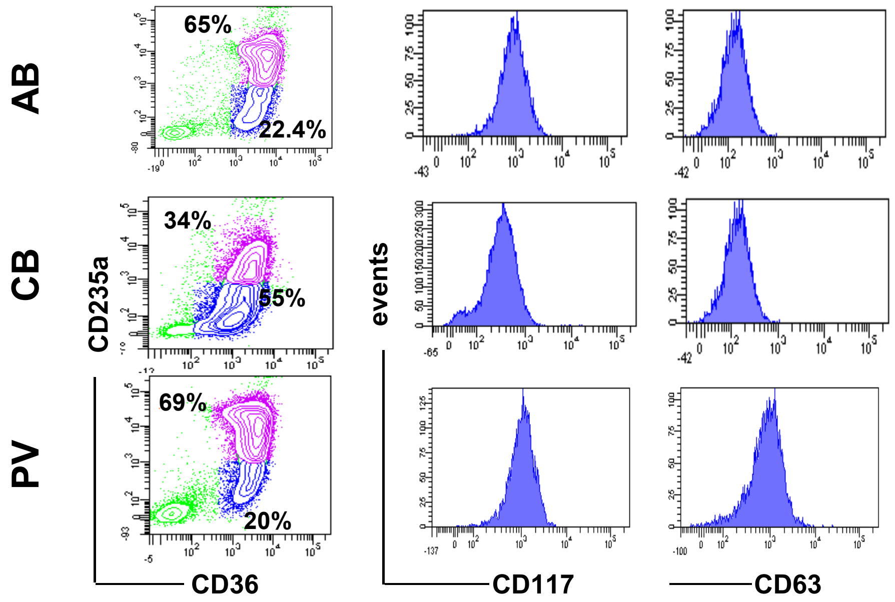

Figure S4

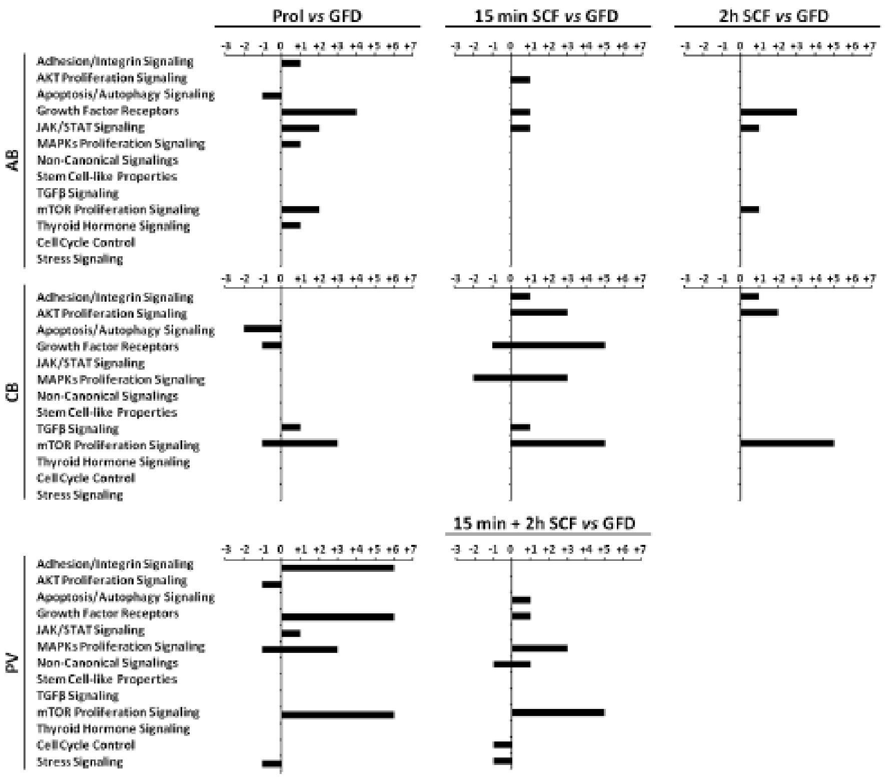

Prol vs GFD
15 min SCF vs GFD
2h SCF vs GFD
15 min + 2h SCF vs GFD
-3 -2 -1 0 +1 +2 +3 +4 +5 +6 +7
AB
CB
PV
Adhesion/Integrin Signaling
AKT Proliferation Signaling
Apoptosis/Autophagy Signaling
Growth Factor Receptors
JAK/STAT Signaling
MAPKs Proliferation Signaling
Non-Canonical Signalings
Stem Cell-like Properties
TGFβ Signaling
mTOR Proliferation Signaling
Thyroid Hormone Signaling
Cell Cycle Control
Stress Signaling

Figure S5

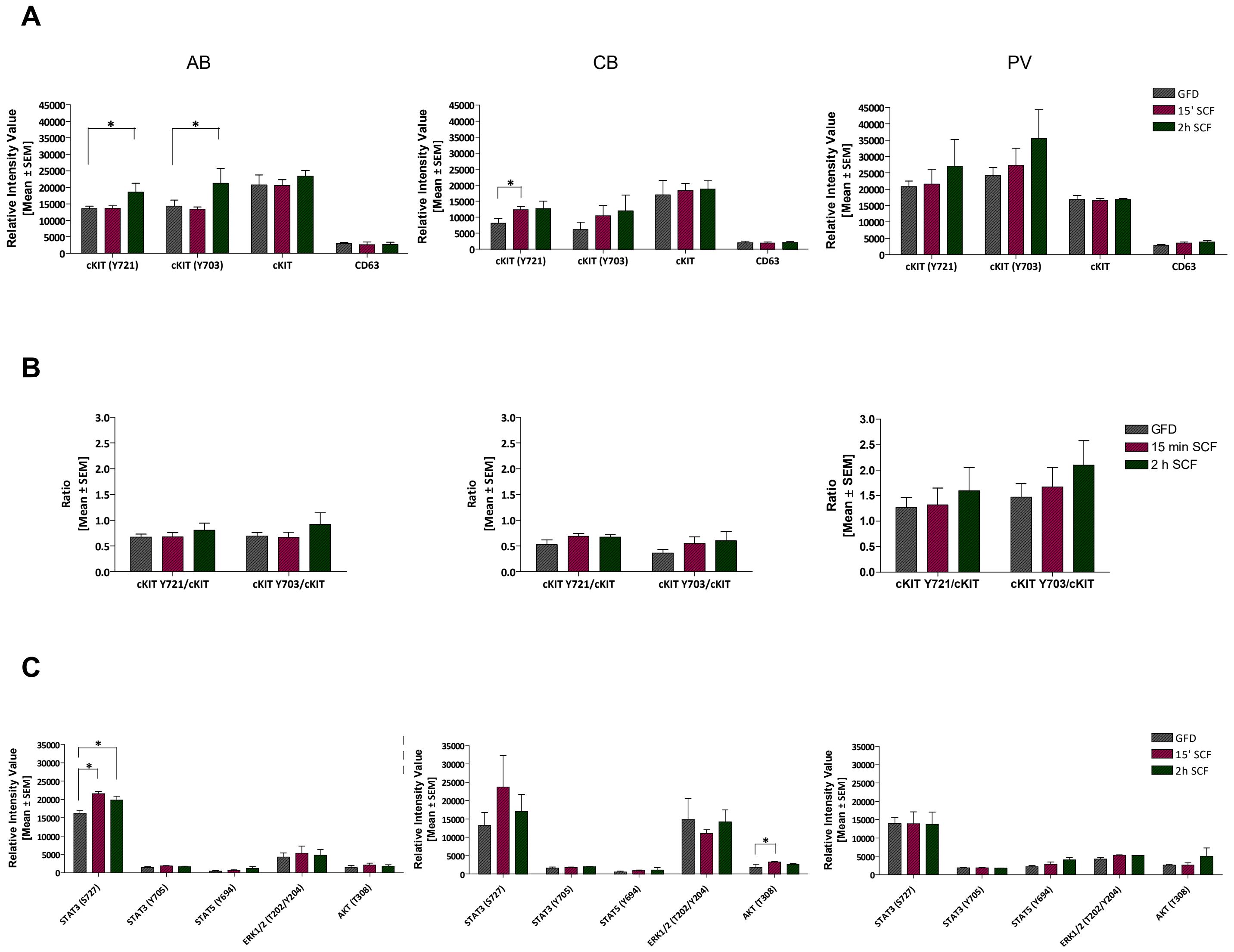
A
AB
CB
PV
GFD
15' SCF
2h SCF
Relative Intensity Value
[Mean ± SEM]
cKIT (Y721)
cKIT (Y703)
cKIT
CD63
B
Ratio
[Mean ± SEM]
15 min SCF
2 h SCF
cKIT Y721/cKIT
cKIT Y703/cKIT
C
STAT3 (S727)
STAT3 (Y705)
STAT5 (Y694)
ERK1/2 (T202/Y204)
AKT (T308)

Figure S6A-C

A

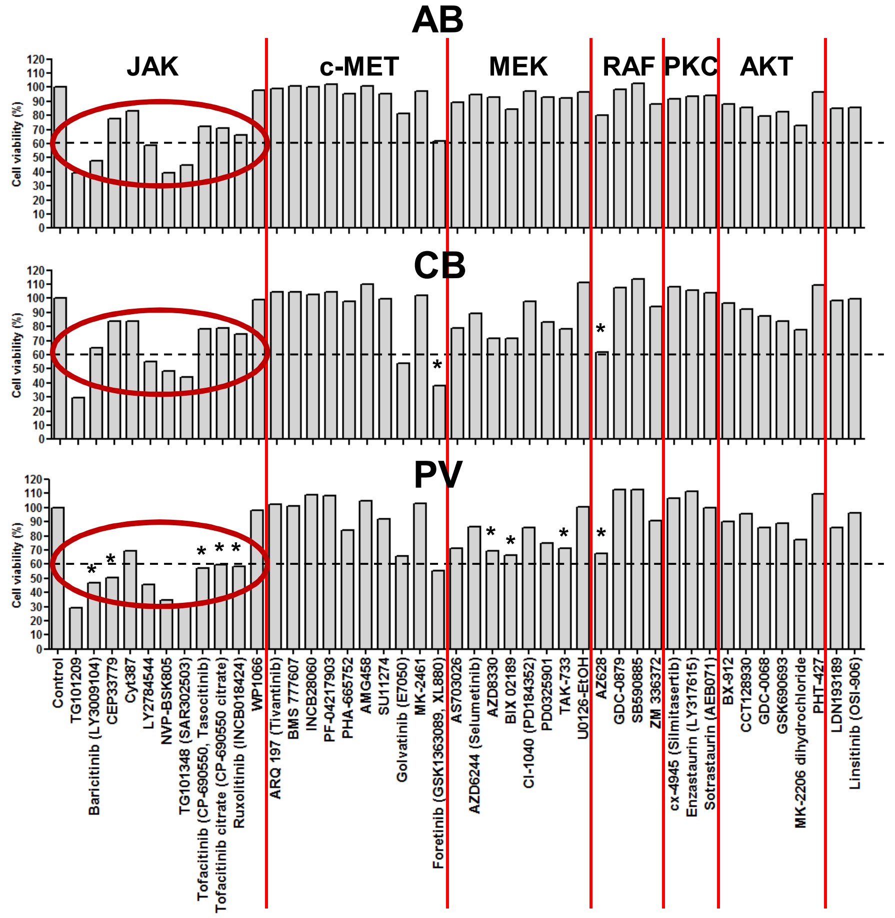

B

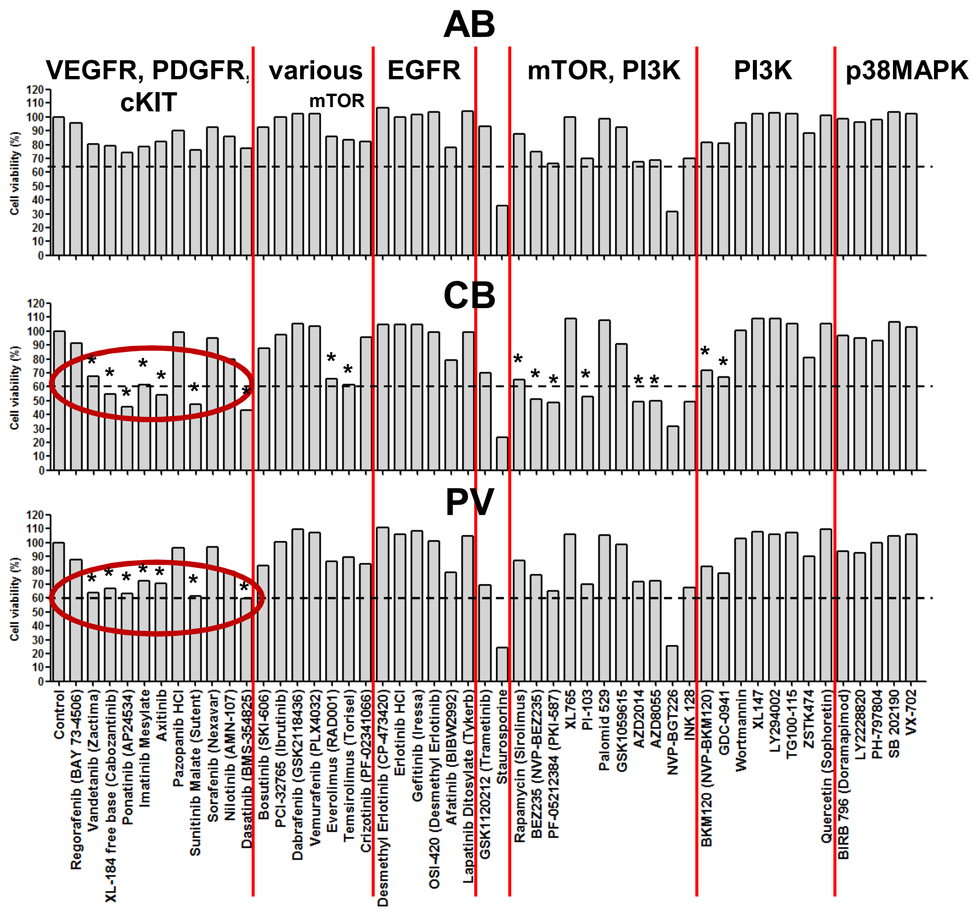

Figure S7A-B

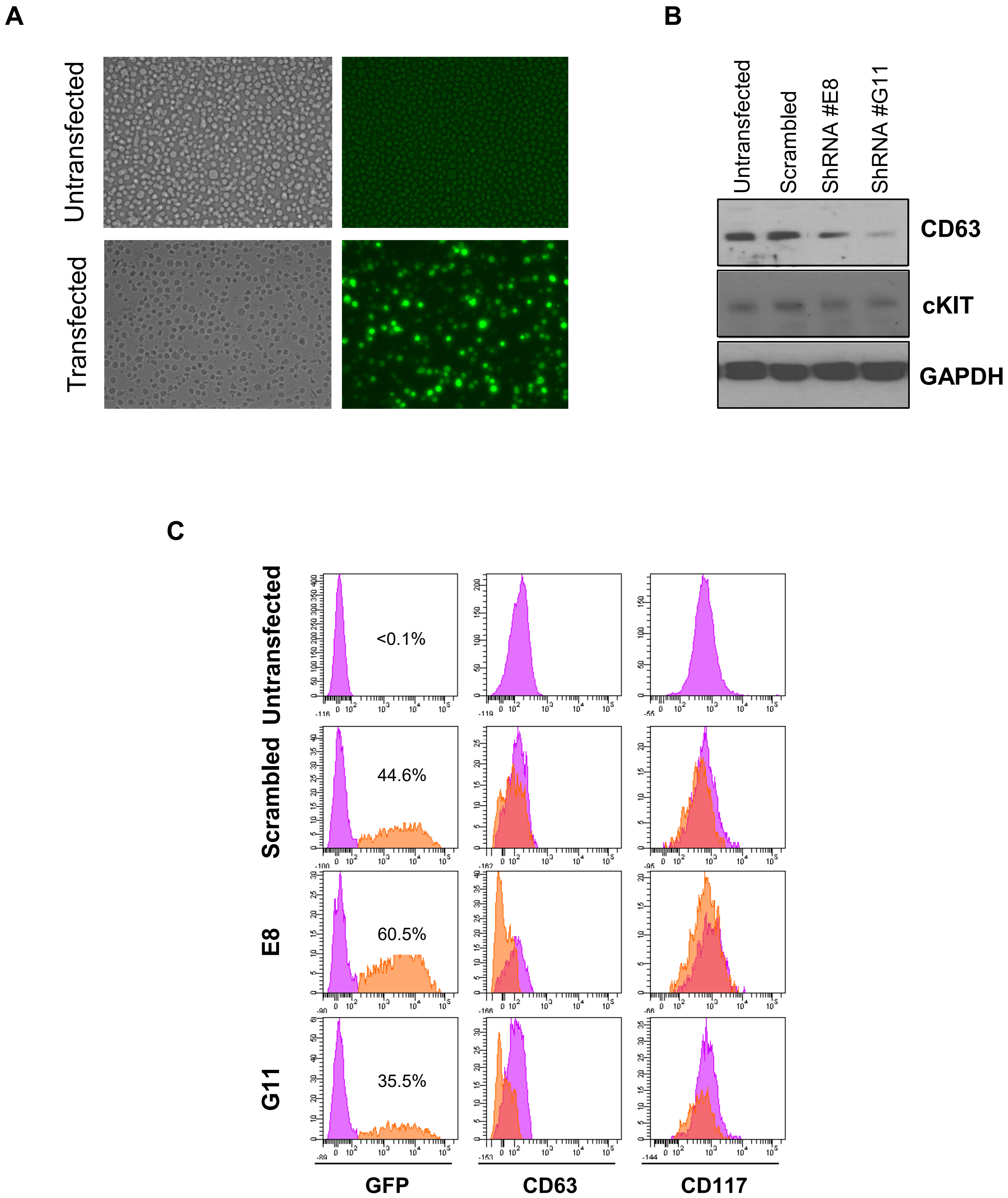
A
Untransfected
Transfected
B
Untransfected
Scrambled
ShRNA #E8
ShRNA #G11
CD63
cKIT
GAPDH
C
Untransfected
Scrambled
E8
G11
<0.1%
44.6%
60.5%
35.5%
GFP
CD63
CD117

Figure S8A-C
